# Active subseafloor microbial communities from Mariana back-arc venting fluids share metabolic strategies across different thermal niches and taxa

**DOI:** 10.1038/s41396-019-0431-y

**Published:** 2019-05-09

**Authors:** Elizabeth Trembath-Reichert, David A. Butterfield, Julie A. Huber

**Affiliations:** 1 Department of Marine Chemistry and Geochemistry, Woods Hole Oceanographic Institution, 266 Woods Hole Road, Woods Hole, MA 02543 USA; 2 Joint Institute for the Study of the Atmosphere and Ocean, University of Washington and NOAA Pacific Marine Environmental Lab, 7600 Sand Point Way NE, Seattle, WA 98115 USA

**Keywords:** Water microbiology, Microbial ecology

## Abstract

There are many unknowns regarding the distribution, activity, community composition, and metabolic repertoire of microbial communities in the subseafloor of deep-sea hydrothermal vents. Here we provide the first characterization of subseafloor microbial communities from venting fluids along the central Mariana back-arc basin (15.5–18°N), where the slow-spreading rate, depth, and variable geochemistry along the back-arc distinguish it from other spreading centers. Results indicated that diverse Epsilonbacteraeota were abundant across all sites, with a population of high temperature Aquificae restricted to the northern segment. This suggests that differences in subseafloor populations along the back-arc are associated with local geologic setting and resultant geochemistry. Metatranscriptomics coupled to stable isotope probing revealed bacterial carbon fixation linked to hydrogen oxidation, denitrification, and sulfide or thiosulfate oxidation at all sites, regardless of community composition. NanoSIMS (nanoscale secondary ion mass spectrometry) incubations at 80 °C show only a small portion of the microbial community took up bicarbonate, but those autotrophs had the highest overall rates of activity detected across all experiments. By comparison, acetate was more universally utilized to sustain growth, but within a smaller range of activity. Together, results indicate that microbial communities in venting fluids from the Mariana back-arc contain active subseafloor communities reflective of their local conditions with metabolisms commonly shared across geologically disparate spreading centers throughout the ocean.

## Introduction

Low-temperature diffusely venting fluids at hydrothermal vents are hot spots of primary productivity in the deep ocean and provide a window into the diverse communities of chemolithoautotrophic bacteria and archaea living in the subseafloor. In these fluids, the concentration of energy sources varies widely depending on the geological setting, chemistry, and fluid dynamics of each system. The majority of studies to date examining diffuse fluids and their microbial communities come from mid-ocean ridges and volcanic arcs around the globe, and have revealed how microbes in these dynamic environments vary with different parameters, including particle attachment versus free-living [1], mafic versus ultramafic host rock [2, 3], geographic distance [4], and local geochemistry and circulation patterns [3–7]. From these studies and others, it is recognized that diffuse vent fluids contain microorganisms representing different mixing regimes and habitats both at and below the seafloor. They have diverse autotrophic and heterotrophic community membership, including mesophilic and moderately thermophilic microaerobic sulfur-metabolizing organisms, as well as higher temperature hydrogen-metabolizing organisms, such as Aquificae and methanogens [1–7]. The presence of these populations is consistent with thermodynamic calculations suggesting sulfide and hydrogen provide the most available energy to microbial communities in deep-sea hydrothermal vents [8, 9].

In addition to characterizing the composition and metabolic repertoire of microbial communities in venting fluids with cultivation and molecular-based approaches, radioisotope [10–13] and stable isotope probing (SIP [2, 14, 15]) techniques can be used to identify, compare, and quantify specific metabolic activities at deep-sea hydrothermal vents. For example, RNA-SIP [16] combines isotope incorporation and sequencing of gene transcripts for organisms that utilize the provided isotopic substrate. This method was first applied at vents to examine subseafloor autotrophs in venting fluids from Axial Seamount [14]. In incubations with ^13^C-bicarbonate addition, a shift in carbon-fixation pathways from reductive tricarboxylic acid (Epsilonbacteraeota [17]) to reductive acetyl-CoA (methanogenic archaea) was observed as incubation temperature increased. Stable isotope experiments have also been used to estimate rates of activity of microbes in venting fluids. For example, FISH-NanoSIMS (fluorescence in situ hybridization-nanoscale secondary ion mass spectrometry) was applied to incubations with hydrothermal vent fluids from the Mid-Atlantic Ridge and Manus Basin using ^13^C-acetate and ^15^N-ammonium to estimate carbon and nitrogen assimilation rates for the heterotrophic microbial community, with results indicating rapid (<8 h) acetate utilization at both 37 and 55 °C [18]. Recently, NanoSIMS analysis of ^13^C-bicarbonate amended fluid incubations carried out at in situ pressures from the East Pacific Rise found highly productive autotrophic communities [15]. Despite only a limited number of experiments in recent years, these tools provide first-order estimates on the activity and metabolic rates of microbial communities in venting fluids and the subseafloor, but more measurements are needed across the full diversity of hydrothermal systems to constrain the contributions of subseafloor and vent microbes to deep-sea biogeochemistry.

In comparison to mid-ocean ridges and volcanic arcs where the bulk of these diffuse vent studies have been carried out, back-arc settings are relatively underexplored and provide a distinct geologic setting capable of supporting hydrothermal vents and subseafloor microbial life [19–21]. The magmas produced at back-arcs are affected by the proximity of the subducting oceanic plate, creating diverse magma chemistry and variation in volatile content. This in turn can affect the composition of hydrothermal fluids, generating a wide range in end-member fluid pH, dissolved gases, and metal concentrations [22–24]. Only a handful of back-arc hydrothermal systems have been explored with a detailed microbiology focus to date, with much of the emphasis on the geologically distinct sedimented Okinawa Trough [21, 25] as well as hydrothermal deposits, fluids, endosymbionts, and plumes from the fast-spreading Manus and Lau Basins [25–33]; therefore, it is difficult to generalize about the resident subseafloor microbial communities along back-arcs.

The central Mariana back-arc was the focus of a recent systematic exploration between 15 and 18.5°N, where a number of new hydrothermal vent sites were discovered along a line of deep basins west of the Mariana arc, resulting in a refined geologic interpretation of the back-arc spreading segments [34, 35]. The central Mariana back-arc is a deep, unsedimented, basalt-hosted, slow-spreading center (25–40 mm/year) that has hydrothermal vent fields from 3200 to 3950 m deep, and is distinct in depth, morphology, and spatial frequency of hydrothermal vent sites from the shallower- and faster-spreading Mariana back-arc south of 13.5°N [23, 34, 35]. Central Mariana back-arc sites also differ from other slow- and ultra-slow-spreading ridges, such as those in the Atlantic and Indian oceans, in that there is no evidence of water-rock interaction with ultramafic sources, usually resulting in venting fluids highly enriched in hydrogen and methane due to serpentinization (e.g., [8]). The slow-spreading rate and great depth also distinguish the Mariana back-arc spreading center from some of the better-studied back-arcs, such as the fast spreading Manus (500–2200 m deep, >100 mm/year) and Lau Basins (~1500–2200 m deep, ~100 mm/year), as well as the most southern end of the Mariana back-arc (~2800 m deep, ~51 mm/year [34]).

Here we provide the first characterization of the microbial community in venting fluids from both previously known and newly discovered vents of the Mariana back-arc basin and place results for subseafloor microbial communities in the context of their unique geologic and geochemical setting, both regionally and globally. We determined the phylogenetic identity, community composition, functional potential, and metabolic activity of microbial communities in venting fluids from five vent fields along the Mariana back-arc, including some of the deepest back-arc vents described to date. In addition, we used single-cell (SIP-NanoSIMS) and single-gene (RNA-SIP) activity experiments targeting subseafloor thermophilic and hyperthermophilic populations. Together, these results provide new insights into the distribution, metabolism, and rates of active microbial assemblages in the warm subseafloor habitats of deep-sea hydrothermal vents along a geologically distinct back-arc basin.

## Materials and methods

### Sample collection

**Fig. 1:**
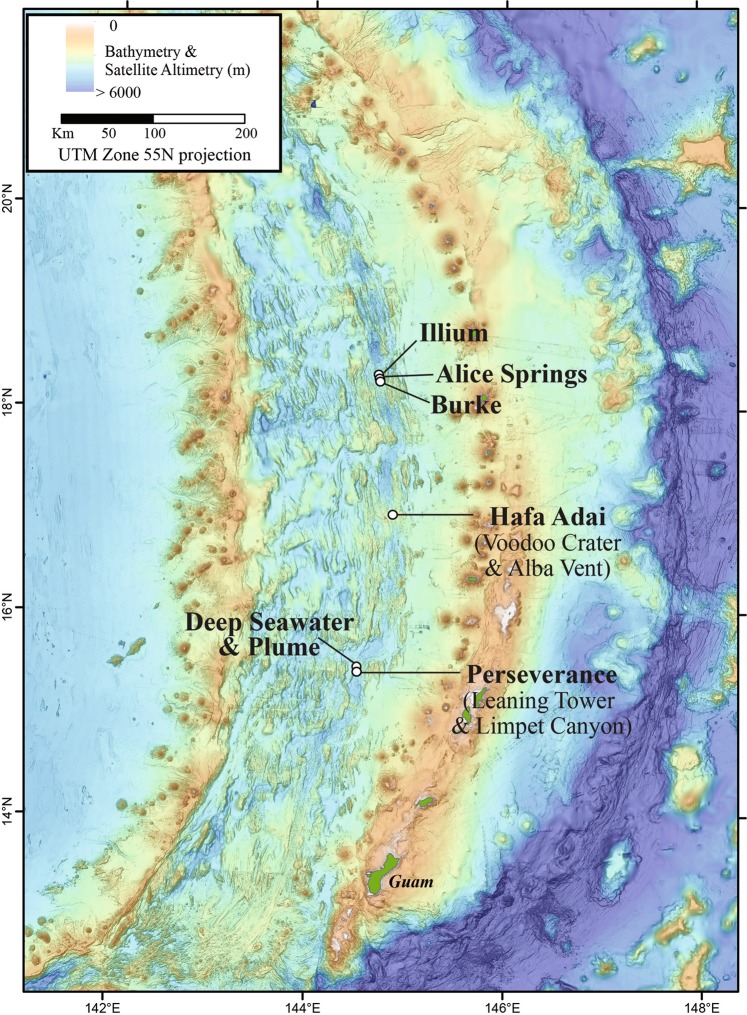
Bathymetric map of vent fields and samples from this study. Map courtesy of Susan Merle and Andrea Bobbit

Diffuse hydrothermal vent fluids were collected along the Mariana back-arc basin in November and December of 2016 on board the R/V *Falkor* using the remotely operated vehicle (ROV) *SuBastian*. Diffuse fluids were sampled at seven distinct sources of low temperature fluid discharge from five different vent fields along the back-arc, from ~15.4 to 18.2°N (Fig. 1, Table 1, Supplemental Table 1). Using the hydrothermal fluid and particle sampler (HFPS;[9]), 3 L of diffuse fluid was pumped at a rate of 100–150 mL/min through a 0.22 μm, 47 mm GWSP filter (Millipore). Temperature was constantly monitored while sampling fluids using an integrated sensor. Oxygen concentration was measured in situ with HFPS by passing fluid through a SeaBird SBE 63 optical oxygen sensor. All filters were preserved in situ with RNALater (Ambion) and frozen at −80 °C as previously described in Akerman et al. [6]. In addition to filters, 4 L of vent fluid was also collected by the HFPS into a large volume bag (LVB) for additional experiments and analyses as described in Fortunato and Huber [14]. LVB water was transferred to incubations using a peristaltic pump and sterile tubing. A CTD (Conductivity, Temperature, Depth) water sampling rosette was used to collect a plume sample approximately 288 m off bottom (3625 m water depth) located near the Perseverance vent field into a 15 L niskin bottle. On the same cast, non-plume, background seawater was also collected ~613 m off bottom at a depth of 3200 m. For both samples, approximately 15 L of water was filtered through a 0.22 μm Sterivex (Millipore) filter on deck, preserved in RNALater, placed at 4 °C for 24 h, and then stored at −80 °C. Fluid samples were also collected for shipboard chemical analysis, including hydrogen sulfide concentration, pH, dissolved hydrogen gas concentration, and dissolved methane gas concentration following methods described in Butterfield et al. [9].

**Table 1:** Location, depth, temperature, cell abundance, geochemistry, and eruption rate (Anderson et al. [34]) for vent sites listed N to S

Site	Vent	Depth (m)	Average temp (max temp, °C)	Cells/ml (±95% conf int)	pH	H_2_S (µM)	H_2_ (µM)	CH_4_ (µM)	Eruption rate (m^3^/year/km)	Oxygen (meas./ bkgd.)
Ilium	Snail Pile Mkr 138	3582	28.2 (32.3)	2.19 × 10^5^ (1.10 × 10^4^)	6.00	13	0.003	1.757	11,739	0.20
Alice Springs	Mkr-131 Snail-001	3611	38.6 (78.0)	1.75 × 10^5^ (7.70 × 10^3^)	5.91	1	0.343	1.696	11,739	0.47
Burke	Snail Pit Mkr-234	3631	40.1 (49.5)	1.76 × 10^5^ (5.90 × 10^3^)	5.85	213	0.003	1.831	11,739	0.41
Hafa Adai	Voodoo Crater-1	3277	34.4 (100.3)	3.16 × 10^5^ (1.70 × 10^4^)	6.05	194	2.380	1.645	31,600	0.26
Hafa Adai	Alba Vent	3295	14.3 (16.4)	2.41 × 10^5^ (1.10 × 10^4^)	6.32	226	1.991	1.111	31,600	0.58
Hafa Adai	Voodoo Crater-2	3277	29.9 (37.4)	4.84 × 10^5^ (1.70 × 10^4^)	6.00	155	2.361	1.561	31,600	0.36
Perseverance	Leaning Tower	3912	35.7 (45.5)	1.10 × 10^5^ (6.50 × 10^3^)	5.61	854	0.238	7.899	3912	0.59
Perseverance	Limpet Canyon	3913	17.7 (21)	1.04 × 10^6^ (3.90 × 10^3^)	5.95	205	0.016	4.004	3912	0.38
Perseverance	In plume	3625	1.6	1.81 × 10^5^ (4.80 × 10^3^)	7.88	0	0.002	0.002	3912	1.00
Perseverance	Background	3200	1.6	1.49 × 10^5^ (4.80 × 10^4^)	7.89	0	0.002	0.002	3912	1.00

### SIP incubations

Vent fluid samples collected by the HFPS LVB were used for cell counts, ^13^C-bicarbonate RNA-SIP incubations (~530 mL each) at either 55 or 80 °C for either 9 or 18 h, and NanoSIMS incubations (~26 mL each, no label, ^2^H_2_O, ^2^H_2_O and ^13^C-acetate, and ^2^H_2_O and ^13^C bicarbonate) at 80 °C for 9 and 18 h. RNA-SIP experiments had a final concentration of 10 mM bicarbonate with 20 mL (900 µmol) of pure H_2_ headspace. When added, NanoSIMS experiments had a final concentration of either 5 mM bicarbonate, 30 μM acetate, and 10% ^2^H_2_O with an overlying atmosphere of 50% N_2_/50% H_2_. Post incubation, RNA-SIP bottles were filtered into 0.22 μm Sterivex filters (Millipore) using a peristaltic pump, preserved in RNALater, and frozen at −80 °C. RNA was extracted from RNA-SIP filters using a mirVana miRNA isolation kit and then gradient preparation, isopycnic centrifugation, and gradient fractionation were performed as described in Lueders et al. [16] and Fortunato and Huber [14]. ^12^C and ^13^C peak separation was confirmed with 16S rRNA reverse transcription-quantitative PCR (RT-qPCR) using the 341F/805R universal primer set [6] and KAPA Biosystems SYBR FAST One Step RT-qPCR (ABI Prism). The ^13^C experiment gradient fraction with the highest 16S rRNA copies was converted to complementary DNA (cDNA) and used for SIP metatranscriptomic library construction. Post incubation, NanoSIMS experiments were preserved with paraformaldehyde to a final concentration of 2 % and stored at 4 °C. Cells from the Voodoo Crater vent NanoSIMS experiments were filtered, washed (to remove unincorporated label and fixative), dehydrated, gold coated, and masses 1, 2, 12, 13, and 26 were collected by NanoSIMS with a primary Cs^+^ beam of 1.5 pA. The Supplemental Methods detail additional cell count, RNA-SIP, and NanoSIMS incubation procedures.

### In situ RNA and DNA

For extraction of nucleic acids from in situ filtered fluids, filters were split in half with one portion used for RNA extraction (mirVana miRNA isolation kit) and the other for DNA extraction (phenol–chloroform as described in Fortunato and Huber [14]). DNA extracted from filters was used for 16S rRNA gene amplicon sequencing with separate archaea [36] and bacteria [37] v4v5 primers (Supplemental Table 2) and metagenome library construction (Nugen Ovation Ultralow Library DR multiplex system). RNA extracted from filters was used for metatranscriptome library construction (Nugen Ovation Complete Prokaryotic RNA-Seq DR multiplex system). See Supplemental Methods for detailed nucleic acid extraction and library preparation protocols.

16S rRNA gene reads were processed using Mothur (v.1.39.5; [38]) and operational taxonomic units (OTUs) were classified with the SILVA v128 database [39]. Metagenome and metatranscriptome paired-end reads were merged and quality filtered using custom Illumina utility scripts [40]. rRNA reads mapping to the SILVA SSU NR database v132 using Bowtie2 v2.2.9 [41] were removed. Percent of reads remaining after this filtering step was included in Supplemental Table 5 for all metatranscriptomes. The resulting reads from in situ metatranscriptomes were then processed with Kallisto v0.43.1 [42] to determine transcripts per million reads mapping to open reading frames from the metagenome of the same vent. Merged reads were assembled for metagenome and RNA-SIP metatranscriptomes using IDBA-UD v1.1 [43]. Assembled contigs from each library were submitted to the DOE Joint Genome Institute Integrated Microbial Genome Metagenomic Expert Review (IMG/MER[44]). IMG-formatted assembly and annotation files were used for manual metagenome-assembled genome (MAG) binning with Anvi'o (v1.2.1) [45] with a minimum contig length of 2500 bp. MAG binning was done manually with Anvi'o (v1.2.1), refined with refineM v0.0.22 [46], and checked with checkM v1.0.9 [47]. Genome trees were constructed using the concat.codon.updated.1.fasta output from Phylosift v1.0.1 [48] and Raxml v8.2.11 [49] with 20 tree runs and 100 bootstraps. Detailed bioinformatics and statistical analyses are described in Supplemental Methods.

## Results

### Site description and sample collection

Diffuse fluids were sampled at seven distinct sources of low temperature discharge from five different vent fields from ~15.4 to 18.2°N on the central Mariana back-arc (Fig. 1, Supplemental Table 1, Supplemental Fig. 1). The Hafa Adai vent field, on the 17°N segment, has a higher eruption rate of 31,600 m^3^/year/km than Perseverance field on the 15.5°N segment (3912 m^3^/year/km; Table 1) or the Ilium, Alice Springs, and Burke fields on the 18.2°N segment (11,739 m^3^/year/km; [34]). These eruption rates are indicative of a robust magma supply that drives vigorous hydrothermal circulation at Hafa Adai, with weaker magma supplies and likely deeper and slightly cooler hydrothermal circulation at the other deep vent fields along the Mariana back-arc. At the three closely spaced northern sites on the 18.2°N segment (Illium, Alice Springs, and Burke), there were no active, high-temperature smokers observed or sampled, although they may be present [35]. All fluid samples in the 18.2°N area were taken in diffuse flow areas with large amounts of animal biomass, including snails, crabs, and shrimp. These vents ranged in average temperature from 28 to 40 °C. At the Alice Springs sampling site (marker 131), there were fluids with temperatures up to 165 °C within 1 m of the low-temperature site sampled, while fluids from Illium and Burke sites were all <50 °C. The two southern vent fields, Hafa Adai at 17°N and Perseverance at 15.5°N, hosted active black smoker chimneys, and one sample from Alba vent of Hafa Adai was collected in 14 °C diffuse flow at the base of a high temperature (239 °C) chimney. Additional samples from the Hafa Adai vent field were collected at Voodoo Crater, a circular feature composed of metal sulfide debris, hosting several sites of active diffuse fluid flow. Diffuse fluids at Voodoo Crater ranging from 30 to 100 °C were collected among or near communities of snails and brachyuran crabs. Finally, at Perseverance, low temperature fluids (36 °C) from a dying chimney (tilted, highly oxidized with no evidence of high temperature flow) named Leaning Tower were sampled, as were fluids from Limpet Canyon, where cracks in the basalt seafloor at the base of the chimney were emanating diffuse fluids with an average temperature of 18 °C (Fig. 1, Supplemental Fig. 1, Table 1). Table 1 gives the properties of diffuse fluids that were sampled for microbiology. Diffuse fluid pH ranged from 5.6 to 6.3 across the back-arc, while hydrogen sulfide concentrations were lowest at Alice Springs (1 μM) and Ilium (13 μM), and from 155 to 226 μM at all other sites, with the exception of Leaning Tower, where the concentration of hydrogen sulfide was 854 μM (Table 1). Hydrogen concentrations were <0.5 μM at all sites except for Hafa Adai, where concentrations were ~ 2 μM; methane concentrations were <2 μM except at Perseverance, where concentrations ranged from 4.9 to 7 μM. Oxygen concentrations in near-bottom deep seawater were 130–140 µmol/L. Oxygen concentrations measured in situ in diffuse fluids ranged from 20 to 59% of local background (Table 1). Cell counts ranged from 1.75 × 10^5^ cells/mL at Alice Springs to 1.04 × 10^6^ at Perseverance (Limpet Canyon; Table 1).

### 16S rRNA gene sequencing diversity

**Fig. 2:**
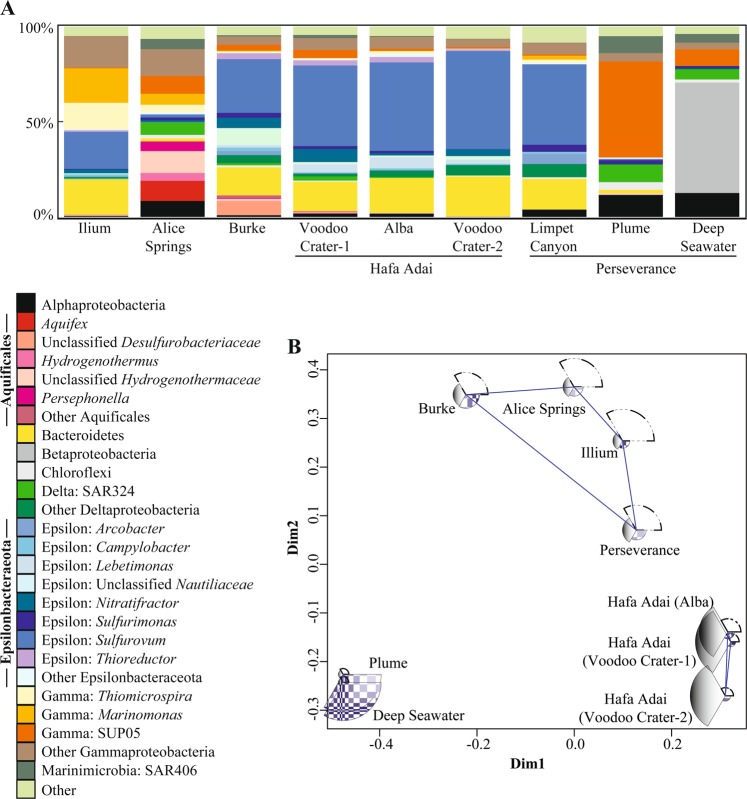
**a** Relative abundance of 16S rRNA gene 97% clustered operational taxonomic units (OTUs) grouped by taxonomy for bacteria for all vent sites and plume and **b** probability of membership in one of three groups (Group 1: checkered, Group 2: shaded, and Group 3: dashed outline) calculated by fuzzy clustering of bacteria. Wedge size indicates probability of membership

For bacteria, Epsilonbacteraeota were the dominant phylum in all vent samples (Fig. 2a), except Alice Springs, where Aquificae dominated. Statistical analyses of bacteria OTU relative abundance were conducted by plotting samples with classical multidimensional scaling and determining fuzzy logic membership in three possible groups. Samples were clearly delineated by plume and background seawater (Group 1), Hafa Adai vents (Group 2), and the rest of the vents (Group 3; Fig. 2b). Groups 1 and 2 were the more distinct and tightly clustered than Group 3. Group 3 was more similar to Group 2 than Group 1. Too few archaea samples were sequenced to perform this analysis (the number of groups must be greater than [*n*]/2 − 1). For archaea, Marine Group I (Thaumarchaeota) dominated all vent samples (50 to 90% of OTUs), followed by Marine Group II (Thermoplasmata; 4 to 46% of OTUs) (Supplemental Fig. 2). Marine Benthic Group A comprised 9% of Illium archaeal sequences, and <2% of the remaining samples. *Archaeoglobi* comprised 28 and 11% of Hafa Adai vents Alba and Voodoo Crater, respectively. *Thermoprotei* comprised 17% of Burke archaeal OTUs. A minor contribution from methanogens was only recovered at Perseverance vent (0.35% *Methanococci*).

### MAG abundance across metagenome, metatranscriptome, and ^13^C-Bicarbonate RNA-SIP metatranscriptomes

**Fig. 3:**
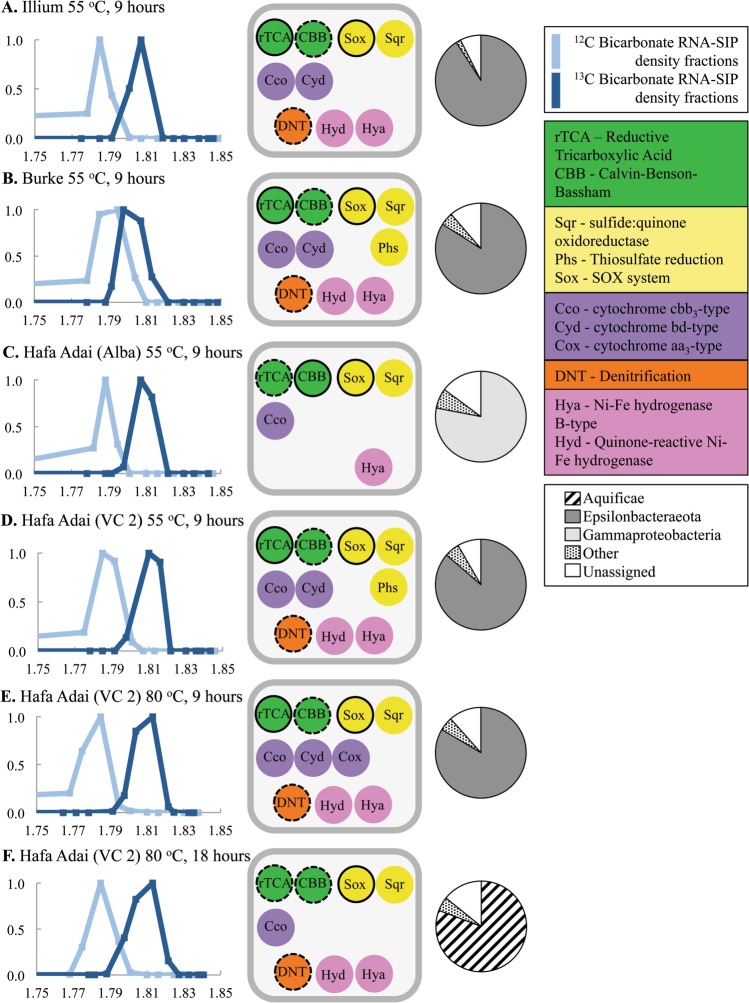
RNA stable isotope probing (RNA SIP) results for **a** Illium 55 °C, 9 h experiment; **b** Burke 55 °C, 9 h experiment; **c** Hafa Adai (Alba) 55 °C, 9 h experiment; **d** Hafa Adai (Voodoo Crater-2) 55 °C, 9 h experiment; **e** Hafa Adai (Voodoo Crater-2) 80 °C, 9 h experiment; and **f** Hafa Adai (Voodoo Crater-2) 80 °C, 18 h experiment. The left column shows density comparison of ^12^C (lighter line) and ^13^C (darker line) RNA-SIP experiments by ratio of 16S rRNA gene reverse transcription-quantitative PCR (RT-qPCR) count over maximum count of all density fractions for all incubations. For 80 °C incubations, the ^13^C-bicarbonate 9 h incubation was also compared to 18 h ^12^C-bicarbonate incubation, since no 9 h ^12^C-bicarbonate incubation was conducted. The middle column shows gene presence in peak ^13^C-RNA-SIP fraction metatranscriptomes. Solid outlined circles indicate complete pathway was observed and dashed outlines indicate partial pathway observed. Single-gene observations have no black outline. The right column shows dominant (>1%) integrated microbial genome (IMG) taxonomic assignment of genes with 30% or greater BLAST identities from RNA-SIP metatranscriptome

Bicarbonate amended RNA-SIP experiments were carried out at all sites at both 55 and 80 °C, except for Illium, where incubations were only carried out at 80 °C due to limited sample recovery (Supplemental Table 3). These temperatures were selected to target resident thermophilic and hyperthermophilic microbes beneath the seafloor and that were flushed out in mixed low-temperature fluids collected from vents [14, 50]. The experiments were carried out identically to those at Axial Seamount [14] for direct comparison of results between a mid-ocean ridge and back-arc spreading center, with the addition of a 9 h time point when sample volume allowed. Overall, more RNA was recovered from 55 °C experiments than 80 °C across all sites. Enough RNA was recovered for centrifugation from six sets of experiments (Supplemental Table 3), and the peaks from the ^13^C fractions were chosen for metatranscriptome library construction, sequencing, and assembly (Fig. 3, Supplemental Tables 4 and 5). In most experiments, Epsilonbacteraeota dominated label uptake (>75% of genes), with the exception of Hafa Adai (Alba) 55 °C 9 h and Hafa Adai (VC2) 80 °C 18 h, where either Gammaproteobacteria or Aquificae dominated, respectively (Fig. 3). Results from these experiments are compared to the in situ filtered fluid metagenomes and metatranscriptomes, as described below.

**Fig. 4:**
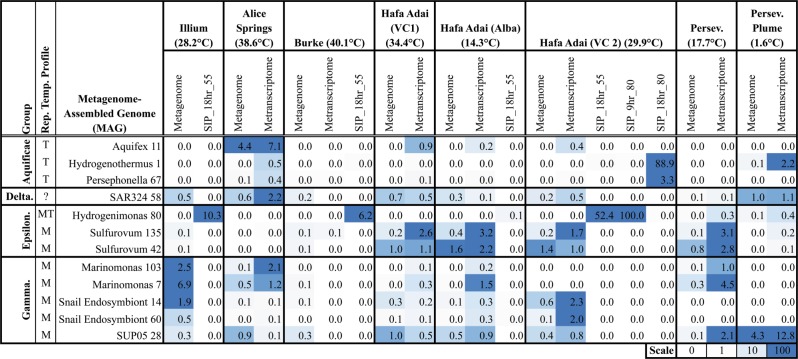
Percent recruitment of reads to each metagenome-assembled genome (MAG) from each in situ metagenome, in situ metatranscriptome, and RNA-stable isotope probing (RNA-SIP) metatranscriptome. Only “active” MAG(s) recruiting >1% of reads from RNA-SIP and/or metatranscriptome samples are included and organized by general taxonomic group based on Phylosift classification. Table should  be read as MAG X recruited Y percent of reads from metagenome sample Z. Rep. Temp. Profile—cultured representative preferred thermal range classification: (M) moderate <35 °C, (MT) moderate thermophile 35–55 °C, (T) thermophile >55 °C. Average fluid temperature is included with the vent name. VC is abbreviated Voodoo Crater and Perseverance was abbreviated Persev. Scale set by percentile to highlight upper and lower ranges where most comparisons were made. White is 10th percentile and below and solid color is 90th percentile

Together, eight in situ filtered fluid metagenomes, six RNA-SIP metatranscriptomes, and seven in situ filtered metatranscriptomes were generated (Supplemental Tables 4 and 5). Binned contigs from in situ filtered metagenome and RNA-SIP metatranscriptome assemblies resulted in 89 MAGs ≥50% complete, with a corrected contamination of <10% (Supplemental Table 6 and 7). 16S rRNA gene relative abundance correlated to MAG read recruitment from in situ metagenome samples, where Epsilonbacteraeota MAGs had the highest read recruitment to Hafa Adai and Perseverance vents and Aquificae MAGs had the highest read recruitment from Alice Springs vent (Fig. 4). The SUP05_28 MAG recruited reads from multiple vent in situ metatranscriptomes, with the highest recruitment from Hafa Adai Voodoo Crater 2, which also had the highest Snail Endosymbiont MAG recruitment (Fig. 4).

Phylogenetic trees for the Epsilonbacteraeota, Aquificae, and Gammaproteobacteria MAGs were constructed (Supplemental Fig. 3–5). Mariana back-arc *Persephonella* MAGs grouped separately from other vent *Persephonella* (Supplemental Fig. 3). Within the Epsilonbacteraeota, the two *Sulfurovum* MAGs (135 and 42) with the highest metatranscriptome recruitment were phylogenetically distinct from the rest of the *Sulfurovum* MAGs generated in this study (Supplemental Fig. 4, star). The SUP05 28 MAG with elevated transcription in vents did not have phylogenetic distinction from other MAGs (Supplementary Fig. 5).

For RNA-SIP experiments, *Hydrogenimonas*_80 was the Epsilonbacteraeota MAG with highest read recruitment in 55 °C experiments, with the highest overall recruitment from Hafa Adai VC2 at 80 °C after 9 h (Fig. 4). By 18 h of the same experiment, almost all of the reads instead mapped to *Hydrogenothermus* and *Persephonella* MAGs belonging to the Aquificae (Fig. 4). The 55 °C RNA-SIP experiment from Hafa Adai Alba did not have high recruitment to any MAGs when mRNA-only reads were used, but most genes were assigned to Gammaproteobacteria in the assembled RNA-SIP metatranscriptome (Fig. 3).

### In situ and RNA-SIP metatranscriptome metabolic pathways

**Fig. 5:**
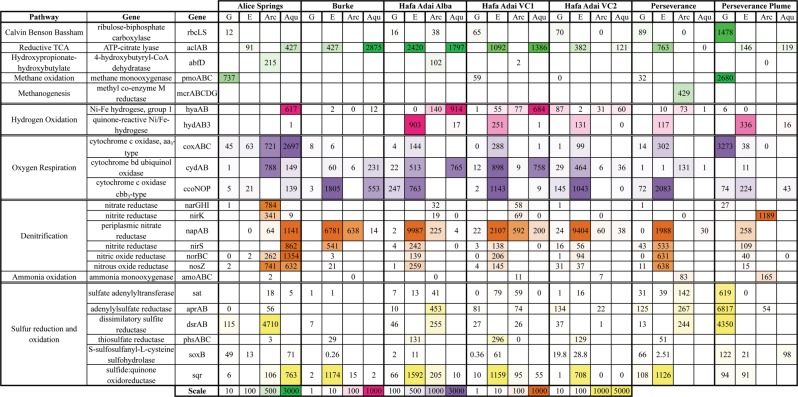
Gene transcription as transcripts per million reads (tpm) at all sites for fluid metatranscriptomes, focusing on carbon fixation, sulfur cycling, nitrogen cycling, hydrogenase, and oxygen utilization genes. G—Gammaproteobacteria, E—Epsilonbacteraeota, Arc—Archaea, Aqu—Aquificae. Colors match pathways in Fig. 3. Scale set by percentile to highlight upper and lower ranges where most comparisons were made. White is 10th percentile and below and solid color is 90th percentile

Key genes involved in pathways for carbon fixation and sulfur, nitrogen, and hydrogen, and oxygen metabolisms were compared by taxonomic assignment across all filtered fluids (Fig. 5) and RNA-SIP experiments for which metatranscriptomes were generated (Fig. 3). The dominant in situ carbon fixation pathway was the reductive tricarboxylic acid (TCA) cycle, performed by Epsilonbacteraeota (all vents) and Aquificae (all vents, except Perseverance). Gammaproteobacteria performing the Calvin–Benson–Bassham cycle dominated carbon fixation in the plume, though there was also a low level of transcription from this process in all vents except Burke. The hydroxypropionate–hydroxybutylate cycle, found in Thermoprotei, also contributed to carbon fixation gene transcripts at Alice Springs and Hafa Adai Alba. While methanotrophs and methanogens were not abundant community members in 16S rRNA gene surveys or MAGs, transcription of methyl co-enzyme M reductase (mcrABCDG) was observed at Perseverance and methane monooxygenase (pmoABC) at Alice Springs and especially in the plume (Fig. 5). PmoABC genes were differentiated from amoABC by taxonomic assignment, where all Gammaproteobacteria genes were from *Methylococcales* and therefore attributed to pmoABC and all archaea genes were from *Thaumarchaeota* and attributed to amoABC.

In the ^13^C-bicarbonate RNA-SIP metatranscriptomes targeting subseafloor autotrophs, the reductive TCA cycle was the only complete carbon fixation pathway recovered, and only from those experiments dominated by Epsilonbacteraeota (Fig. 3). A partial reductive TCA cycle was recovered from the 18 h experiment at Voodoo Crater 2 at 80 °C, where Aquificae dominated (Fig. 3). Partial Calvin–Benson–Bassham cycles were recovered in all RNA-SIP metatranscriptomes, with genes for the complete cycle present in the Hafa Adai Alba experiment, which was dominated by a Gammaproteobacteria related to the scaly snail endosymbiont (Supplemental Fig. 5). No methanogenesis genes were recovered in RNA-SIP experiments.

Two types of hydrogenase transcripts were detected in the in situ metatranscriptomes, including hya-type (hyaAB) and quinone-reactive Ni/Fe-type (hydA3B3). Hya-type was predominately transcribed by Aquificae at Alice Springs and Hafa Adai vents (Fig. 5). It was also present in all RNA-SIP metatranscriptomes (Fig. 3). The quinone-reactive Ni/Fe-type was predominately transcribed by Epsilonbacteraeota in all vents except Alice Springs and Burke. This form of hydrogenase was also detected in all of the RNA-SIP metatranscriptomes, except for the experiment from Hafa Adai Alba, where Gammaproteobacteria dominated.

Cytochrome *c* oxidase cbb3-type (cco) and cytochrome bd ubiquinol oxidase (cyd) gene transcripts were the most abundant form across most vents, with cco primarily affiliated with Epsilonbacteraeota (Campylobacterales) and to a lesser degree, Aquificae (Desulfurobacteriales; Fig. 5). The cyd form was uniquely utilized by Nautiales and Aquificales. Cytochrome *c* oxidase aa3-type (cox) was highly transcribed by archaea (Thermoprotei) and Aquificae (Aquificales) at Alice Springs and Gammaproteobacteria in the plume. In the RNA-SIP experiments, the cbb3 and bd types were present in all Epsilonbacteraeota dominated incubations, while the Alba and the Hafa Adai VC2 80 °C 18 h experiments only had the cbb3 type. All three types (including the low-affinity aa3 type) were present in the 9 h 80 °C Hafa Adai VC2 incubation (Fig. 3).

Transcripts for nitrate reduction were abundant in all vents, with the napAB nitrate reductase/nirS nitrite reductase as the most dominant forms (Fig. 5). At Alice Springs, these genes were transcribed by Aquificae (Aquificales and Desulfurobacteriales napAB only), while at all the other sites they were mainly attributed to Epsilonbacteraeota (Campylobacterales) and to a lesser degree, archaea (Thermoprotei). Alice Springs and the plume also had high transcription of the narGHI (Thermoprotei) and nirK (Thaumarchaeota) forms, attributed to archaea. Complete denitrification (including norBC and nosZ) was dominated by Epsilonbacteraeota at Hafa Adai and Perseverance vents and by Aquificae and archaea at Alice Springs. A partial denitrification pathway was recovered in all RNA-SIP experiments, with the exception of Hafa Adai Alba where Gammaproteobacteria dominated (Fig. 3).

With respect to sulfur cycling, sulfate reduction (sat, aprAB, dsrAB) transcripts affiliated with Gammaproteobacteria (SUP05 and Endosymbiont) dominated plume samples, while transcripts affiliated with Archaea (Archaeoglobi/Thermoprotei) dominated at Alice Springs, Hafa Adai, and Perseverance. Transcripts for sulfur oxidation via the sox pathway (denoted by the key enzyme soxB) were detected at all sites except for Burke, mainly attributed to Gammaproteobacteria (Thiotrichales, SUP05) and Epsilonbacteraeota (Campylobacterales), although soxB transcripts were attributed to Aquificae (Aquificales) at Alice Springs (Fig. 5). The highest sulfur-related transcription in vents was from the sulfide:quinone oxidoreductase (sqr) gene, which catalyzes quinone by taking electrons from sulfide [51]. This transcript was mainly affiliated with Epsilonbacteraeota (Campylobacterales) at all vents except for Alice Springs, where it was detected in both archaea (Thermoprotei) and Aquificae (Aquificales; Fig. 5). The gene thiosulfate reductase (phsABC) was highly transcribed in all vents, except Alice Springs, and also affiliated with Epsilonbacteraeota (Nautiliales). In RNA-SIP metatranscriptomes, the complete Sox pathway was recovered from all experiments except for Burke, and sulfide:quinone oxidoreductase (sqr) was detected in all experiments (Fig. 3). Transcripts for thiosulfate reduction (phs) were only present in 55 °C incubations at Hafa Adai Voodoo Crater 2 and Burke.

### SIP NanoSIMS experiments

SIP-NanoSIMS incubations were prepared onboard in triplicate with either no label, ^2^H_2_O (general activity tracer), ^2^H_2_O and ^13^C-acetate (heterotrophy tracer), or ^2^H_2_O and ^13^C-bicarbonate (autotrophy tracer). Incubations were conducted at 9 and 18 h time points at 80 °C with fluids from Burke, Alba, Voodoo Crater, and Leaning Tower, for a total of 96 incubations. Since the Voodoo Crater RNA-SIP experiments were successful at 80 °C, these NanoSIMS experiments were chosen for comparability and as the most likely to demonstrate activity. When cell counts were averaged across triplicate samples for each experimental condition, there were higher cell densities at the later time points and no observable growth effect from ^2^H_2_O addition, unlike carbon additions, which yielded both the highest and lowest cell counts across all incubations (Supplemental Fig. 6).

**Fig. 6:**
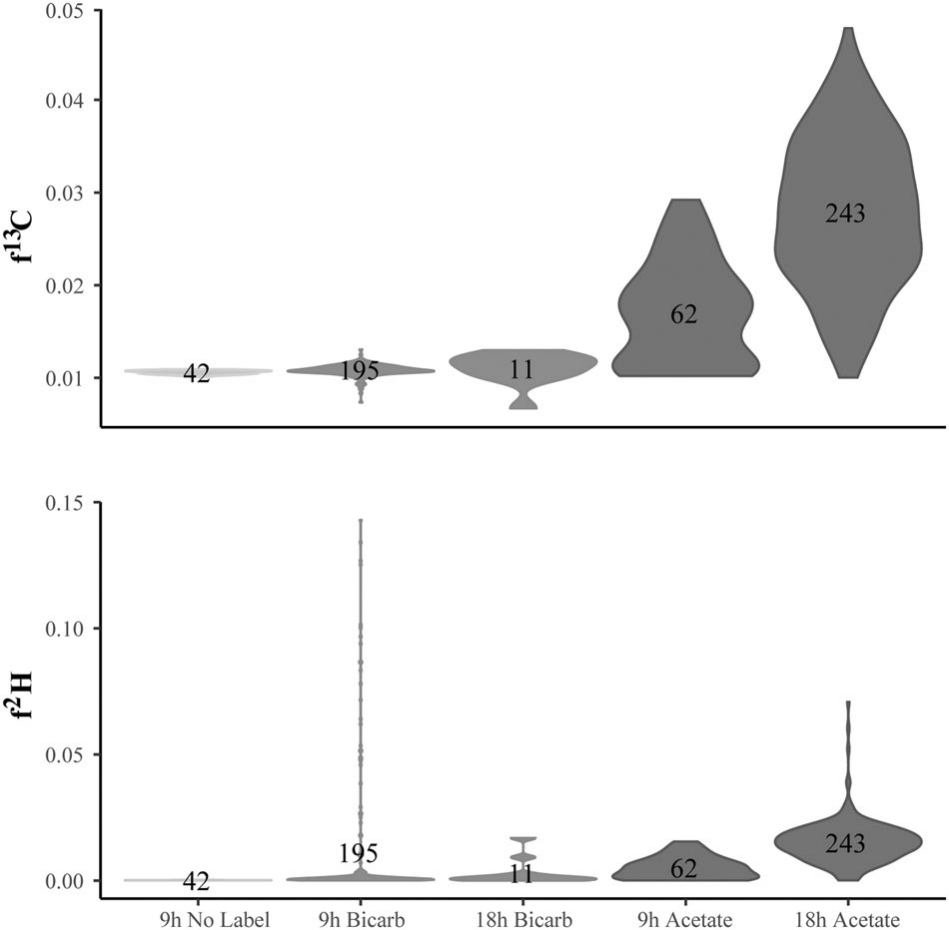
Violin plots of single-cell NanoSIMS (nanoscale secondary ion mass spectrometry) measurements of the fractional abundance of ^13^C and ^2^H across all ^13^C-added conditions from Hafa Adai (Voodoo Crater 2) with no label condition for comparison. Number of regions of interests (cells) is presented on each violin plot

Equal volumes were combined from each triplicate and filtered together for NanoSIMS analysis. From NanoSIMS analysis, the 18 h ^13^C-acetate + ^2^H_2_O incubation (*n* = 243 regions of interest) had the highest average ^13^C enrichment (~2.6× natural abundance) and the largest range in ^13^C enrichment (none to 4.5× natural abundance; Fig. 6). The 9 h ^13^C-acetate + ^2^H_2_O incubation had the second highest number of cells analyzed (*n* = 62) and the second highest average enrichment (~0.6x natural abundance). In contrast, more cells were observed  from the 9 h ^13^C-bicarbonate + ^2^H_2_O condition (*n* = 40) than the 18 h ^13^C-bicarbonate + ^2^H_2_O (*n* = 2), and only the 9 h ^13^C-bicarbonate + ^2^H_2_O condition showed any cells with enrichment above natural abundance (Fig. 6). When observing activity via ^2^H uptake from ^2^H_2_O, the highest absolute enrichment was from the 9 h ^13^C-bicarbonate incubation, and the second highest from the 18 h ^13^C-acetate incubation (Fig. 6).

Carbon and hydrogen biomass generation times were calculated for 9 and 18 h incubations with ^13^C-acetate + ^2^H_2_O, since only these incubations had statistically significant ^13^C ion counts (see Supplemental Methods). Assuming no in situ acetate, ^13^C 9 h generation times ranged from 1.7 to 6.7 days and 18 h generation times ranged from 1.7 to 6.2 days (Supplemental Fig. 7). Assuming an upper estimate of 30 μM in situ acetate, ^13^C 9 h generation times ranged from 0.6 to 2.8 days and 18 h generation times ranged from 1.7 to 6.2 days. ^2^H 9 h generation times ranged from 1.1 to 8.6 days and 18 h from 1.3 to 6.1 days. There was more agreement between the ^13^C-acetate and ^2^H_2_O assessments of biomass generation time when assuming 0 μM in situ acetate concentration (Supplemental Fig. 7). Also, ^2^H generation times appear slightly faster at the 9 h time point and more at parity with ^13^C generation times after 18 h incubation, suggesting a slower population may be captured in the 18 h time point that was not captured in the 9 h time point.

16S rRNA gene sequencing of the NanoSIMS incubations was compared to the in situ filtered vent fluid samples to assess diversity post incubation. Of the 44 OTUs with >1% relative abundance across all NanoSIMS incubations (Supplemental Table 8), six met the same relative abundance threshold in vent samples, classified as *Sulfurovum* [1], *Desulfurobacterium* [3], *Aquifex* [1], and *Pseudoalteromonas* [1]. The remaining OTUs were only detected in the NanoSIMS incubations and belong to a variety of taxa, including Actinobacteria, Betaproteobacteria, and Bacteroidia. These groups are most likely sequencing contaminants, supported by their lack of representation in any of the vent or plume samples, as well as their similarity to common reagent contaminants [52] and physiologies unlikely to be successful in thermophilic temperature regimes. It is therefore most likely that the anabolically active cells detected by NanoSIMS are of the four taxa also found in the vent fluids, and in particular the four Aquificae OTUs, as the only hyperthermophilic group among the vent-specific OTUs. In experiments with either no carbon source added or the acetate addition (Supplemental Table 8), the relative abundance of Aquificae (*Desulfurobacterium*) increased with time, with the 9 h experiments composed of 25–31% Aquificae and the 18 h experiments composed of 45–92% Aquificae. In experiments with ^13^C-bicarbonate addition, Aquificae were <0.01% at either time point and Epsilonbacteraeota and Gammaproteobacteria were more likely the active community members.

## Discussion

In this study, we assessed microbial community composition and activity at both previously known and newly discovered vents along the Mariana back-arc, the deepest back-arc vents studied to date. Much of the previous work along back-arcs examined microbial community composition in sulfide deposits and microbial mats, which are known to be distinct habitats from venting fluids [32, 53]. Along the Mariana back-arc, the only other microbiological studies were carried out at the lower latitude vents from ~13°N, where the back-arc segment more closely resembles a fast-spreading axis [21, 34] than the slower spreading observed in the central back-arc of this study. 16S rRNA gene profiling was used to examine microbial community structure at this lower latitude location in mats, active sulfides, inactive sulfides, and venting fluids, including fluids sampled from shallow (~10 mbsf) holes generated with seabed drills [54]. While no -omic or stable isotope assays were carried out at these sites, 16S rRNA gene surveys (Snail and Pika vents) indicated that fluids and microbial mats were dominated by Zetaproteobacteria [54–57], a group of organisms rarely detected in the vent fluids sampled in our study. Given this, we focus our discussion on the similarities and distinctions between the sites sampled along the central Mariana back-arc in this study and compare results to other spreading ridges where similar datasets exist, thus placing our results in a global context of the distribution and activity of subseafloor microbial communities at deep-sea hydrothermal vents.

Along the central Mariana back-arc, bacterial 16S rRNA gene analyses revealed that the northern-most (18.2°N, Illium, Alice Springs, and Burke) and southern-most (15.5°N, Perseverance) vents had a more similar community structure compared to the vents on the middle segment (17°N, Alba and Voodoo Crater from Hafa Adai). This was also apparent in metagenomic binning and transcript profiles, with Epsilonbacteraeota belonging to *Sulfurovum* as the most abundant and active group at almost all sites, especially Hafa Adai. Furthermore, two phylogenetically distinct *Sulfurovum* MAGs had elevated read recruitment from Hafa Adai metatranscriptomes, suggesting a potential sub-genus preference for these vent conditions. The exception to this was Alice Springs, where instead Aquificae dominated in relative abundance and activity. Such a distribution may be linked to overall vent field activity, where the central Hafa Adai vent field had many actively growing metal sulfide chimneys, and was much hotter and more vigorous than the northern (Alice Springs–Burke–Illium) or southern (Perseverance) vent fields. The level of hydrothermal activity on any spreading axis is related to the underlying geology and tectonic setting, and while the three back-arc segments sampled for this study share the same basaltic host rock, their tectonic settings differ: the Hafa Adai vent field on the 17°N segment has a more robust magmatic contribution than the northern and southern sites, as well as a considerably higher eruption rate [34, 35]. This is reflected in higher measured diffuse flow temperatures (100 °C) and hydrogen concentrations (~2 µM) at Hafa Adai. Hydrogen sulfide concentrations were higher at Hafa Adai and Perseverance fields than at both Ilium and Alice Springs on the 18.2°N segment, where hydrogen and hydrogen sulfide were extremely low (<13 µM H_2_S and <0.4 µM H_2_). At Alice Springs, transcripts for the lower affinity oxidase (aa3-type, cox) were also highly expressed. In contrast, at all other vents, transcripts for the higher affinity oxidase (cbb3-type, cco) were dominant and carried by Epsilonbacteraeota. This suggests Alice Springs’ low sulfide conditions favor microaerophilic Aquificae over the *Sulfurovum* found in high abundance at all other vents. A distinction was also seen in sulfur metabolism between these two groups, where transcripts affiliated with the Epsilonbacteraeota for thiosulfate utilization (phs) were highly transcribed at all sites except for Alice Springs, where instead the transcripts for sulfide oxidation (sqr) were abundant and affiliated with Aquificae. Despite these differences in which organisms were present across the back-arc, transcripts for oxygen respiration, hydrogen oxidation, and denitrification were found at all the sites. This suggests that similar metabolisms were utilized across disparate taxonomic and thermal groups. The only metabolism with a highly restricted presence along the back-arc was methanogenesis. Methanogens were uniquely recovered at relatively low abundance in 16S rRNA gene profiles and mcrA transcripts at the Perseverance vent. The presence of methanogens at Perseverance correlates to the highest methane concentrations for all diffuse vent samples (up to 8 µM), but no MAGs were recovered for this group.

RNA-SIP experiments targeted the thermophilic and hyperthermophilic autotrophs of the subseafloor that are difficult to capture in mixed venting fluids. Cultured representatives of *Sulfurovum,* the most abundant organism detected in many fluid samples, are mesophilic and are not known to grow at the temperatures of our experiments. Instead, the known thermophilic Epsilonbacteraeota *Hydrogenimonas* dominated across all 55 °C experiments and sites. While there is only one cultured representative from this genus, it has the ability to grow microaerobically while carrying out hydrogen oxidation, as well as anaerobically using hydrogenotrophy coupled to sulfur reduction or denitrification [58]. These are all metabolisms detected in our experiments, thus lending this organism a level of metabolic flexibility ideal for the RNA-SIP experimental conditions. In contrast, many of the hyperthermophilic groups detected via the -omics methods have more restricted metabolisms, such as obligate need for oxygen (e.g., *Aquifex* [51]), the inability to use nitrate or sulfur (e.g., *Hydrogenothermus* [59]), or an obligate need for anoxic conditions (e.g. methanogens). Despite this, *Hydrogenothermus* were successful in the longer (18 h) 80 °C experiment where *Hydrogenimonas* was first present in the shorter (9 h) incubation, suggesting *Hydrogenothermus* may be able to operate in an anaerobic mode not yet described in cultured isolates. In vents, oxygen from deep seawater is added to vent fluid in the subseafloor, making it available for aerobic or micro-aerobic metabolisms. In situ oxygen measurements yielded concentrations that are 20–60% of local ambient bottom seawater; therefore, oxygen was available in all the fluids characterized in this study. It is possible that in the RNA-SIP experiments, oxygen was consumed quickly and concentrations were too low for Aquificae to be successful from Alice Spring fluids, whereas at other sites, organisms such as *Hydrogenimonas* and *Hydrogenothermus* were successful under the lower oxygen experimental conditions. The low-standing stock of methanogens at Perseverance and their need for strictly anoxic conditions likely explains the lack of any methanogenesis detected in the RNA-SIP experiments. Finally, while there are no cultured representatives for comparison to the Snail Endosymbiont 60 MAG, genes for carbon fixation, aerobic respiration, and hydrogenases were present in the RNA-SIP metatranscriptome assembly. The success of Snail Endosymbiont 60 MAG in 55 °C incubations suggested they have a similar thermotolerance to *Hydrogenimonas*, but not as high as *Hydrogenothermus*.

Together, SIP-NanoSIMS and RNA-SIP results from Voodoo Crater suggest that 80 °C incubations were an extreme condition where only a small, predominantly Aquificae, portion of the in situ microbial community remained active, while the *Sulfurovum* dominant in the source vent fluid were selected against. While ^13^C-bicarbonate uptake was minimal in NanoSIMS experiments, a limited portion of the population had the highest overall rates of activity across all experiments as determined by ^2^H uptake (Fig. 6). Differences in volume could explain why the 80 °C RNA-SIP ^13^C-bicarbonate experiment from Voodoo Crater enriched for Aquificae, whereas the NanoSIMS experiment did not. The larger volume RNA-SIP experiment would both take longer to reach 80 °C, therefore providing less temperature shock, and have a larger supply of additional substrates that could support mixotrophic growth during the incubation. When combined with 16S rRNA gene sequencing results, Aquificae most likely incorporated the acetate in the extreme temperatures (80 °C) of the NanoSIMS incubations. Across all experiments, Aquificae were the most active portion of the microbial assemblage in the higher temperature incubation regime, and were able to sustain growth at 80 °C in both autotrophic and heterotrophic modes, as well as in incubations with no labeled carbon substrate.

^13^C-acetate SIP-NanoSIMS was also conducted on back-arc fluids from Manus Basin and mid-ocean ridge fluids from the Mid-Atlantic Ridge (Menez-Gwen [60]). After incubation with ^13^C-acetate, the Manus Basin fluids incubated at 37 °C for 10 h shifted to being dominated by *Marinobacter* (Gammaproteobacteria) and Menez Gwen fluids incubated at 55 °C for 8 h shifted to being dominated by *Nautilia*, with an overall observed enrichment of a minor heterotrophic population rather than a shift of autotrophs to acetate utilization as a supplemental carbon source. A similar shift from *Sulfurimonas* to *Thioreductor* with increased temperature was observed in ^13^C-bicarbonate NanoSIMS incubations with fluids from Crab Spa on the mid-ocean ridge East Pacific Rise [15]. Estimates of biomass turnover time ranged from 0.7 to 1.7 days based on Crab Spa ^13^C-bicarbonate incorporation rates. These times overlap with the lower end of our conservative estimates for ^13^C-acetate and ^2^H generation times at surface pressure and higher temperature (80 °C), suggesting heterotrophic carbon incorporation and overall biomass generation may happen at similar rates to autotrophy in vent systems, with the caveat of differences in incubation pressure and temperature for these two analyses. The East Pacific Rise experiments also determined the highest rates of carbon fixation with nitrate and hydrogen amendments, supporting the importance of hydrogenotrophic denitrification for chemolithoautotrophic microorganisms in the subseafloor of hydrothermal vents from both a fast-spreading mid-ocean ridge and slow-spreading back-arc basin.

In a comparable study of vent fluid microbiology from a back-arc (Manus Basin), Epsilonbactereota belonging to the genera *Sulfurovum* and *Sulfurimonas* were consistently the most abundant taxa throughout all water samples, with warmer diffuse flow vents also hosting Aquificae and cooler, more plume-like samples hosting the Gammaproteobacteria SUP05 [32]. Both *Sulfurimonas* and SUP05 are commonly found in venting fluids and plumes from other spreading centers and volcanic arcs including the Mariana Arc [4], Axial Seamount [6, 14, 61], and Lau Basin [33], but SUP05 was in relatively low abundance and *Sulfurimonas* was rarely detected in the Mariana back-arc vents sampled in this study. These groups were similarly absent from the hydrogen-rich fluids of the ultra-slow-spreading Mid-Cayman Rise [2, 62], suggesting that the mixing regime of fluids at both the Mariana back-arc and Mid-Cayman Rise may not favor these aerobic and microaerophilic organisms known to thrive just above the seafloor where fluids mix with oxygen-replete deep seawater. The reason for the lack of these common groups of vent organisms in Mariana back-arc or Mid-Cayman Rise fluids is not known.

At the mid-ocean ridge volcano Axial Seamount, metagenomic, metatranscriptomic, and RNA-SIP datasets exist for direct comparison to our work along the Mariana back-arc. Diverse Epsilonbacteraeota and Aquificae play an important role in autotrophic nitrogen and sulfur cycling at both sites, with carbon fixation dominated by the reductive TCA cycle in Epsilonbacteraeota and Aquificae, as well as the Calvin–Benson–Bassham cycle in Gammaproteobacteria. However, an important difference was observed with methanogenic archaea, which are key subseafloor autotrophs at 55 °C and above at Axial Seamount [5, 14, 63], but were only detected in the in situ metatranscriptome at Perseverance on the back-arc. Hydrogen concentrations in diffuse vents sampled for this study were <2.5 µM, significantly less than diffuse vents at Axial Seamount (1–30 µM [9]) as well as many other slow-spreading ridges, such as the Mid-Cayman Rise, where hydrogen concentrations reach 3 mM and methanogens are abundant [2]. Methane concentrations were also <8 μM, near the low end of concentrations seen at Axial and most ultra-slow-spreading centers, thus lower methanogenic activity is not surprising from these Mariana back-arc vents. In 55 °C experiments from Axial and Mariana back-arc, both sites showed high activity of Epsilonbacteraeota carrying out hydrogenotrophic denitrification, as well as hydrogen and sulfur oxidation, while utilizing the reductive TCA cycle for carbon fixation (Figs. 4, 5). However, different organisms were present in the experiments, with *Hydrogenimonas* and relatives of scaly snail endosymbionts in Mariana back-arc experiments and *Nautilia* and *Caminibacter*, as well as some moderately thermophilic methanogens belonging to the *Methanothermococcus*, in the experiments at Axial. While different subseafloor autotrophs existed at these different sites, possibly due to a restricted geographic distribution (e.g., *Hydrogenimonas*), our results indicate the same autotrophic metabolisms linked to hydrogen-, nitrate-, and sulfur-based metabolisms dominate when methanogens are not present, highlighting the ability of diverse taxa to exploit the best energy sources in the subseafloor of deep-sea hydrothermal vents.

## Conclusion

Uncovering principles that govern microbial distribution and activity is challenging in any environment, especially in the subseafloor habitat at deep-sea hydrothermal vents where variable fluid flow patterns in a heterogeneous rocky matrix result in highly complex environments that are difficult to delineate. Here we have taken the approach of comparing methods of assessing in situ microbial diversity and activity with SIP incubations under a range of conditions targeting thermophilic members of the vent microbial community. Overall, we found distinct patterns of microbial diversity that correlated to the underlying geological and geochemical differences along the central Mariana back-arc. Of the common autotrophic taxa detected along the Mariana back-arc, Aquificae were more abundant and active in less reducing fluids (low H_2_S and H_2_) and Epsilonbacteraeota in more reducing fluids (high H_2_S and/or H_2_), but both performed similar metabolisms, including hydrogen oxidation, denitrification, and sulfur/thiosulfate oxidation. Some of these organisms, such as *Sulfurovum*, appear universally distributed across many deep-sea hydrothermal vent habitats, whereas others, such as *Hydrogenimonas* and *Hydrogenothermus*, may have a more restricted distribution or occupy a narrow environmental niche that has been infrequently observed to date. More studies of back-arc and other slow-spreading centers are needed to understand controls on the distribution and activity of these important subseafloor autotrophic players, as they represent understudied members of the community with few cultured isolates when compared to methanogens and Aquificales. Finally, the single-cell resolution provided by SIP-NanoSIMS experiments demonstrated that while net carbon anabolism may be similar under our elevated temperature incubation regime, a small number of highly active cells dominated autotrophic conditions and a larger number of less active cells dominated heterotrophic conditions. Together, our results provide new insights into the distribution and activity of subseafloor microbial communities at deep-sea hydrothermal vents, and as more studies combine -omics with experimental incubations, further constraints on the impact of these communities on deep-sea biogeochemistry will be possible.

## Supplementary information

Supplemental Text

Supplemental Figure 1

Supplemental Figure 2

Supplemental Figure 3

Supplemental Figure 4

Supplemental Figure 5

Supplemental Figure 6

Supplemental Figure 7

Supplemental Table 1

Supplemental Table 2

Supplemental Table 3

Supplemental Table 4

Supplemental Table 5

Supplemental Table 6

Supplemental Table 8

Supplemental Table 7
