# Supplementary material for: Active subseafloor microbial communities from Mariana back-arc venting fluids share metabolic strategies across different thermal niches and taxa
Source: ISME J. 2019 May 9;13(9):2264–79. doi: 10.1038/s41396-019-0431-y (PMC6775965; doi:10.1038/s41396-019-0431-y)
Supplement: Supplementary file 1 — Supplemental Text [file 41396_2019_431_MOESM1_ESM.docx]

**Supplemental Methods**

*Cell counts*

To quantify microbial biomass using epifluorescent microscopy, vent fluid was collected into the LVB and preserved in labeled scintillation vials, 2 x 18 mL, with 1.8 mL 37% formaldehyde. Vials were mixed via shaking after adding fixative, sealed with electrical tape, and stored at 4^o^C. On land, cells were quantified using DAPI and 100X objective (Supplemental Table 1).

*RNA Stable Isotope Experiments*

RNA-SIP methods followed the protocol of Fortunato and Huber (2). Briefly, vent fluid was collected from the HFPS Large Volume Bag (LVB) to fill evacuated 500 mL bottles to a volume of 530 mL. A volume of 8.83 mL of either 12C (Sigma, St. Louis, MO, USA) or 13C sodium bicarbonate (Cambridge Isotope Laboratories, Tewksbury, MA, USA) was added to each bottle for a final concentration of 10 mM bicarbonate. After bicarbonate addition, 1-2 mL of 10% HCl was added until the fluid had a pH < 6.5 to ensure the SIP incubation was similar to vent conditions. Then 20 mL (~900 μmoles) of 99.99% H_2_ gas was added to each bottle for a concentration of ~20 μM H_2_ in solution. Bottles were incubated lying on their sides in an incubator at either 55^o^C or 80^o^C for either 9hr or 18hr. Post incubation, bottles were filtered into 0.22 μm Sterivex filters (Millipore) using a peristaltic pump, preserved in RNALater and frozen at -80ºC.

RNA was extracted using the mirVana miRNA isolation kit (Ambion), with an additional lysis step using RNA PowerSoil beads (MoBio, Carlsbad, CA, USA), and DNase treated using the Turbo-DNase kit (Ambion). Gradient preparation, isopycnic centrifugation, and gradient fractionation were performed as described in Lueders et al. (3). For each gradient sample, 5.1 ml of CsTFA (~2 g ml^-1^, GE Healthcare Life Sciences, Piscataway, NJ, USA), 185 µl formamide, 750 ng RNA (two samples were run with ~200 ng RNA due to low extraction yield), and 1 ml gradient buffer solution (0.1 M Tris-HCl, 0.1 M KCl, 0.1 mM EDTA) were first mixed in a 15 ml tube. Once mixed, the refractive index was measured for each sample to ensure a median density of ~1.80 g ml^-1^. Samples were then loaded into 4.9 ml OptiSeal tubes (Beckman Coulter, Brea, CA, USA), placed into a VTI 65.2 vertical rotor (Beckman Coulter) and spun at 37,000 rpm at 20 ºC for 64 h using an Optima L-80 XP ultracentrifuge (Beckman Coulter). Each gradient was fractionated into 12 tubes of approximately 410 µl each and the refractive index of each fraction was measured. RNA was precipitated with isopropanol and the pellet was washed with 70% ethanol as described in Lueders et al. (3). RNA concentration of each fraction was determined using the RiboGreen quantification kit (Invitrogen) and a Gemini XPS plate reader (Molecular Devices, Sunnyvale, CA, USA). 12C and 13C peak separation was confirmed with 16S rRNA RT-qPCR using the 341F/805R universal primer set (4) and KAPA Biosystems SYBR FAST One Step RT-qPCR (ABI Prism). The density fraction with the highest number of 16S rRNA copies in the ^13^C-experiment was used to construct metatranscriptomic libraries using the methodology of Fortunato and Huber (2). Briefly, double stranded cDNA was constructed using SuperScript III First-strand synthesis system (Invitrogen, Grand Island, NY, USA) and mRNA second strand synthesis module (NEB, Ipswich, MA, USA). Double stranded cDNA was sheared to a fragment size of 175bp using a Covaris S-series sonicator (Woburn, MA, USA). SIP transcriptomic library construction was completed using the Ovation Ultralow Library DR multiplex system (Nugen, San Carlos, CA, USA) following manufacturer instructions. Ribosomal RNA was not removed before construction of libraries.

*SIP-Nanoscale Secondary Ion Mass Spectrometry Experiments and Data Processing*

Sealed balch tubes were prepared on land in the anaerobic chamber with 10% labeled substrates. For bicarbonate, 175 μl of a 600 mM stock solution with a 12C:13C ratio of 10 to 1 was added to each tube, while for acetate, 63 μL of a 10 mM stock solution of acetate with a 12C:13C ratio of 10 to 1 was added to each tube. With ~20 mL of vent fluid added to each tube, this resulted in a final concentration of approximately 5 mM DIC and 30 μM acetate. 2 mL of 99.99% ^2^H_2_O was also added to each tube for a final concentration of 10%. Each Balch tube was gassed with N_2_ before shipment. After vent fluid was added to each tube from the LVB using a peristaltic pump, 2 bars of hydrogen was added, giving an overlying atmosphere of 50% N_2_/50% H_2_. Triplicate tubes for each experimental treatment (no label, only ^2^H_2_O, ^2^H_2_O and ^13^C Acetate, and ^2^H_2_O and ^13^C Bicarbonate) were incubated at 80°C for 9 and 18 hours. To end the experiment, the headspace was released and 1 mL of 40% paraformaldehyde was added to each tube for a final concentration of 2%. Each tube was shaken gently and stored at 4°C. Cell counts were performed on each replicate of all SIP-NanoSIMS incubations from the Voodoo Crater vent at Hafa Adai as above. 1 mL from each replicate was combined and filtered onto a 0.22 μm polycarbonate membrane (Millipore), washed 3X with 3XPBS (Invitrogen), and dehydrated with Ethanol. Dried filters were coated with 10 nm gold prior NanoSIMS analysis. The remaining sample (~15 ml from each replicate, ~45 ml total) was filtered onto a 0.22 μm Sterivex for 16S rRNA gene sequencing. DNA was extracted using the phenol-chloroform, amplicon library, and sequence analysis methods described above for filtered in situ samples.

Masses 1, 2, 12, 13, and 26 were collected for all samples along with the secondary electrons using a NanoSIMS-50L (Cameca) at the Caltech Microanalysis Center. A focused primary Cs^+^ beam of 1.5pA was used for data collection after 175pA pre-sputtering of ~15 min. Eight frames of 512 x 512 px at 3300 cts/px were collected for each sample. Data was processed using Look@NanoSIMS software (5). Individual ion image frames were merged and aligned using the ^12^C^14^N ion image to correct for drift during acquisition. Cell-based regions of interest (ROIs) were determined by “interactive thresholding” with the ^12^C^14^N ion image. Final ion images and counts per ROI were calculated by summation of ion counts for each pixel over all scans. Isotopic enrichment was calculated as the fold increase over the non-labeled condition for ^2^H and ^13^C uptake in cellular biomass, per cell (ROI). Single cell generation times for ^2^H and ^13^C incorporation were calculated as per Trembath-Reichert et al. (6) for cells with statistically significant ^13^C fractional abundances (greater than or equal to two times the calculated fractional abundance shot noise) using the equations 1, 2, and 3. ^2^H counts are not as statistically robust due to extremely low natural abundance of ^2^H, but generation times are provided for the cells where the ^13^C fractional abundances passed this threshold for comparison. A water assimilation value (a_w_) of 0.6 was used based on Zhang et al. 2009. ^13^C incorporation was calculated for a range assuming vent fluids had no in situ acetate (30 μM total C, at 0.1 F_label_) and 30 μM in situ concentration (60 μM total at 0.05 F_label_), where 35 μM acetate was the upper range found for venting fluids (7). F_label_ was assumed to be 0.1 for ^2^H since natural abundance ^2^H is insignificant compared to this labeling strength. F_nat_ is the natural abundance of ^13^C (0.0119) and ^2^H (0.0001157) for their respective equations. T_final_ is the length of the incubation (9 hrs or 18 hrs).

^2^H-based generation rate [Eq 1]: ^2^μ= [−ln(1 − [^2^F_final_ − ^2^F_nat_]/a_w_[^2^F_label_ − ^2^F_nat_])]/T_final_

^13^C-based generation rate [Eq 2]: ^13^μ= [−ln(1− [^13^F_final_ − ^13^F_nat_]/[^13^F_label_ −^13^F_nat_])/ T_final_

Generation time [Eq 3]: τ =μ^−1^

*Metagenome, Metatranscriptome, and 16S rRNA gene Library Preparation and Sequencing*

The 47mm PES 0.22 filters (Millipore) were first cut in half with sterile scissors, with half used for DNA extraction and half used for RNA extraction, according to Fortunato and Huber (2). For DNA extraction, the DNA filter was first rinsed with sterile PBS to remove RNAlater and then was extracted using a phenol-chloroform method adapted from Crump et al. (8) and Zhou et al. (9). 16S rRNA genes were amplified in tripplicate using v4v5 primers for archaea (10) and bacteria (11) for 35 cycles. Amplicon sequencing was performed with an Illumina MiSeq at the W.M. Keck sequencing facility at the Marine Biological Laboratory, Woods Hole, MA. For metagenomics library construction, DNA was sheared to a fragment size of 175 bp using a Covaris S-series sonicator and a library prepared using the Ovation Ultralow Library DR multiplex system (Nugen, San Carlos, CA) following manufacturer instructions.

RNA was extracted using the mirVana miRNA isolation kit (Ambion, Grand Island, NY, USA) with an added bead-beating step using RNA PowerSoil beads (MoBio, Carlsbad, CA, USA). A total volume of 100 µl was extracted and was then DNase treated using the Turbo-DNase kit (Ambion), purified, and concentrated using the RNAeasy MinElute kit (Qiagen, Hilden, Germany). Ribosomal RNA removal, cDNA synthesis, and metatranscriptomic library preparation was carried out using the Ovation Complete Prokaryotic RNA-Seq DR multiplex system (Nugen) following manufacturer instructions. Prior to library construction, cDNA was sheared to a fragment size of 175 bp using a Covaris S-series sonicator. Metagenome and metatranscriptome sequencing was performed on an Illumina NextSeq 500 at the W.M. Keck sequencing facility at the Marine Biological Laboratory, resulting in an average read length of 151 bp.

*Sequencing Analysis*

For 16S rRNA genes, sequencing reads were processed using mothur (v.1.39.5) (12) and OTUs were classified with the SILVA v128 database (13). OTUs greater than or equal to 0.1% of a sample were retained and used for figure generation in R (14) with *cluster* (15). A distance matrix of bacteria OTUs was computed using Bray-Curtis dissimilarity with *vegan* (16). This matrix was then ordinated using classical multidimensional scaling with the R function *cmdscale* and clustered with the maximum allowable number of clusters (3) and membership exponent (1.6), as described in the following tutorial <http://cc.oulu.fi/~jarioksa/opetus/metodi/sessio3.pdf>.

For metagenomes and metatranscriptomes, paired-end reads were merged and quality filtered using custom Illumina utility scripts (17). Merged reads were assembled for metagenome and RNA SIP metatranscriptomes using IDBA-UD v1.1 (18) with maxk set to sequence length (150 bp), mink and step size of 20 bp. Assembly statistics were computed for each metagenome with quast v4.5 (19). Assembled contigs from each library were submitted to the DOE Joint Genome Institute Integrated Microbial Genome Metagenomic Expert Review (IMG/MER). IMG formatted assembly and annotation files were used for manual MAG binning with Anvio (v1.2.1) (20) with a minimum contig length of 2500 bp. IMG annotation files were converted to anvio format using in-house python scripts. Bins were refined with refineM v0.0.22 (21) and MAG statistics were calculated with checkM v1.0.9 (22). MAGs were re-imported into Anvio to produce MAG percent recruitment across samples using the bin_output script available here https://github.com/edgraham/BinSanity/tree/master/utils.

Genome trees were constructed using the concat.codon.updated.1.fasta output from Phylosift v1.0.1 (23) and Raxml v8.2.11 (24) with 20 tree runs and 100 bootstraps. Genomes were selected from IMG, JGI, and supplementary information from (25), (26), (27), and (28).

To remove rRNA reads from the metatranscriptomes, reads were mapped to the SILVA SSU NR database 132 (13) using Bowtie2 v 2.2.9 (29) with a local alignment and default settings where only the unmapped reads were retained. Mapping to the LSU database was also done, but less than 1% of total reads mapped for all samples. The resulting “mRNA-only” reads were then processed with Kalliso v0.43.1 (30) to determine transcripts per million reads mapping to ORFs from the paired metagenome. RNA-SIP metatranscriptomes did not contain enough reads for a robust comparison with Kallisto and are instead presented as presence/absence from the metatranscriptome assembly. GFF files were converted from contig format to ORF format using the script gff2seqfeatures.py found here https://github.com/ctSkennerton/scriptShed. The file used to generate Figure 5 is included as a supplemental dataset and contains the higher resolution taxonomic information referenced in the text that there was not space to include in Figure 5.

All raw sequencing data is available through SRA under project number: PRJNA454888 with biosample numbers for each site listed in Supplemental Table 1. All metagenome assemblies are available through the JGI GOLD database under GOLD study ID: Gs0129105. NCBI Genome submission accession numbers for MAGs provided in Supplemental Table 6.

**References**

1. Butterfield DA, Roe KK, Lilley MD, Huber JA, Baross JA, Embley RW, et al. Mixing, reaction and microbial activity in the sub-seafloor revealed by temporal and spatial variation in diffuse flow vents at axial volcano. In American Geophysical Union (AGU); 2004 [cited 2018 Sep 26]. p. 269–89. Available from: http://www.agu.org/books/gm/v144/144GM17/144GM17.shtml

2. Fortunato CS, Huber JA. Coupled RNA-SIP and metatranscriptomics of active chemolithoautotrophic communities at a deep-sea hydrothermal vent. ISME J [Internet]. 2016 Aug 12 [cited 2018 Apr 30];10(8):1925–38. Available from: http://www.nature.com/articles/ismej2015258

3. Lueders T. Stable Isotope Probing of Hydrocarbon-Degraders. In: Handbook of Hydrocarbon and Lipid Microbiology [Internet]. Berlin, Heidelberg: Springer Berlin Heidelberg; 2010 [cited 2018 Apr 30]. p. 4011–26. Available from: http://link.springer.com/10.1007/978-3-540-77587-4_312

4. Takahashi S, Tomita J, Nishioka K, Hisada T, Nishijima M. Development of a Prokaryotic Universal Primer for Simultaneous Analysis of Bacteria and Archaea Using Next-Generation Sequencing. Bourtzis K, editor. PLoS One [Internet]. 2014 Aug 21 [cited 2018 May 12];9(8):e105592. Available from: http://dx.plos.org/10.1371/journal.pone.0105592

5. Polerecky L, Adam B, Milucka J, Musat N, Vagner T, Kuypers MMM. Look@NanoSIMS - a tool for the analysis of nanoSIMS data in environmental microbiology. Environ Microbiol [Internet]. 2012 Apr [cited 2018 Jul 7];14(4):1009–23. Available from: http://doi.wiley.com/10.1111/j.1462-2920.2011.02681.x

6. Trembath-Reichert E, Morono Y, Ijiri A, Hoshino T, Dawson KS, Inagaki F, et al. Methyl-compound use and slow growth characterize microbial life in 2-km-deep subseafloor coal and shale beds. Proc Natl Acad Sci U S A [Internet]. 2017 Oct 31 [cited 2018 Oct 2];114(44):E9206–15. Available from: http://www.pnas.org/lookup/doi/10.1073/pnas.1707525114

7. Lang SQ, Butterfield DA, Schulte M, Kelley DS, Lilley MD. Elevated concentrations of formate, acetate and dissolved organic carbon found at the Lost City hydrothermal field. Geochim Cosmochim Acta [Internet]. 2010 Feb 1 [cited 2018 Jul 7];74(3):941–52. Available from: https://www.sciencedirect.com/science/article/pii/S001670370900684X

8. Crump BC, Kling GW, Bahr M, Hobbie JE. Bacterioplankton community shifts in an arctic lake correlate with seasonal changes in organic matter source. Appl Environ Microbiol [Internet]. 2003 Apr 1 [cited 2018 May 2];69(4):2253–68. Available from: http://www.ncbi.nlm.nih.gov/pubmed/12676708

9. Zhou J, Bruns MA, Tiedje JM. DNA recovery from soils of diverse composition. Appl Environ Microbiol [Internet]. 1996 Feb [cited 2018 May 2];62(2):316–22. Available from: http://www.ncbi.nlm.nih.gov/pubmed/8593035

10. Topçuoğlu BD, Stewart LC, Morrison HG, Butterfield DA, Huber JA, Holden JF. Hydrogen Limitation and Syntrophic Growth among Natural Assemblages of Thermophilic Methanogens at Deep-sea Hydrothermal Vents. Front Microbiol [Internet]. 2016 Aug 5 [cited 2018 May 1];7:1240. Available from: http://journal.frontiersin.org/Article/10.3389/fmicb.2016.01240/abstract

11. Huse SM, Young VB, Morrison HG, Antonopoulos DA, Kwon J, Dalal S, et al. Comparison of brush and biopsy sampling methods of the ileal pouch for assessment of mucosa-associated microbiota of human subjects. Microbiome [Internet]. 2014 Feb 14 [cited 2018 May 1];2(1):5. Available from: http://www.microbiomejournal.com/content/2/1/5

12. Kozich JJ, Westcott SL, Baxter NT, Highlander SK, Schloss PD. Development of a Dual-Index Sequencing Strategy and Curation Pipeline for Analyzing Amplicon Sequence Data on the MiSeq Illumina Sequencing Platform. Appl Environ Microbiol [Internet]. 2013 Sep 1 [cited 2018 May 1];79(17):5112–20. Available from: http://www.ncbi.nlm.nih.gov/pubmed/23793624

13. Quast C, Pruesse E, Yilmaz P, Gerken J, Schweer T, Yarza P, et al. The SILVA ribosomal RNA gene database project: improved data processing and web-based tools. Nucleic Acids Res [Internet]. 2012 Nov 27 [cited 2018 May 1];41(D1):D590–6. Available from: http://academic.oup.com/nar/article/41/D1/D590/1069277/The-SILVA-ribosomal-RNA-gene-database-project

14. R Core Team. R: A Language and Environment for Statistical Computing [Internet]. Vienna, Austria; 2017. Available from: https://www.r-project.org/

15. Maechler M, Rousseeuw P, Struyf A, Hubert M, Hornik K. cluster: Cluster Analysis Basics and Extensions. 2017.

16. Oksanen J, Blanchet FG, Friendly M, Kindt R, Legendre P, McGlinn D, et al. vegan: Community Ecology Package [Internet]. 2017. Available from: https://cran.r-project.org/package=vegan

17. Eren AM, Vineis JH, Morrison HG, Sogin ML. A Filtering Method to Generate High Quality Short Reads Using Illumina Paired-End Technology. Jordan IK, editor. PLoS One [Internet]. 2013 Jun 17 [cited 2018 Jul 7];8(6):e66643. Available from: http://dx.plos.org/10.1371/journal.pone.0066643

18. Peng Y, Leung HCM, Yiu SM, Chin FYL. IDBA-UD: a de novo assembler for single-cell and metagenomic sequencing data with highly uneven depth. Bioinformatics [Internet]. 2012 Jun 1 [cited 2018 May 1];28(11):1420–8. Available from: http://www.ncbi.nlm.nih.gov/pubmed/22495754

19. Gurevich A, Saveliev V, Vyahhi N, Tesler G. QUAST: quality assessment tool for genome assemblies. Bioinformatics [Internet]. 2013 Apr 15 [cited 2018 May 3];29(8):1072–5. Available from: http://www.ncbi.nlm.nih.gov/pubmed/23422339

20. Eren AM, Esen ÖC, Quince C, Vineis JH, Morrison HG, Sogin ML, et al. Anvi’o: an advanced analysis and visualization platform for ‘omics data. PeerJ [Internet]. 2015 Oct 8 [cited 2018 May 1];3:e1319. Available from: https://peerj.com/articles/1319

21. Parks DH, Rinke C, Chuvochina M, Chaumeil P-A, Woodcroft BJ, Evans PN, et al. Recovery of nearly 8,000 metagenome-assembled genomes substantially expands the tree of life. Nat Microbiol [Internet]. 2017 Nov 11 [cited 2018 May 1];2(11):1533–42. Available from: http://www.nature.com/articles/s41564-017-0012-7

22. Parks DH, Imelfort M, Skennerton CT, Hugenholtz P, Tyson GW. CheckM: assessing the quality of microbial genomes recovered from isolates, single cells, and metagenomes. Genome Res [Internet]. 2015 Jul 1 [cited 2018 May 1];25(7):1043–55. Available from: http://www.ncbi.nlm.nih.gov/pubmed/25977477

23. Darling AE, Jospin G, Lowe E, Matsen FA, Bik HM, Eisen JA. PhyloSift: phylogenetic analysis of genomes and metagenomes. PeerJ [Internet]. 2014 Jan 9 [cited 2018 May 2];2:e243. Available from: https://peerj.com/articles/243

24. Stamatakis A. RAxML version 8: a tool for phylogenetic analysis and post-analysis of large phylogenies. Bioinformatics [Internet]. 2014 May 1 [cited 2018 May 2];30(9):1312–3. Available from: http://www.ncbi.nlm.nih.gov/pubmed/24451623

25. Fortunato CS, Larson B, Butterfield DA, Huber JA. Spatially distinct, temporally stable microbial populations mediate biogeochemical cycling at and below the seafloor in hydrothermal vent fluids. [cited 2018 Mar 19]; Available from: https://onlinelibrary.wiley.com/doi/pdf/10.1111/1462-2920.14011

26. Anderson RE, Reveillaud J, Reddington E, Delmont TO, Eren AM, McDermott JM, et al. Genomic variation in microbial populations inhabiting the marine subseafloor at deep-sea hydrothermal vents. Nat Commun [Internet]. 2017 Dec 24 [cited 2018 Mar 19];8(1):1114. Available from: http://www.nature.com/articles/s41467-017-01228-6

27. Sheik CS, Anantharaman K, Breier JA, Sylvan JB, Edwards KJ, Dick GJ. Spatially resolved sampling reveals dynamic microbial communities in rising hydrothermal plumes across a back-arc basin. ISME J [Internet]. 2015 Jun 9 [cited 2018 Apr 30];9(6):1434–45. Available from: http://www.ncbi.nlm.nih.gov/pubmed/25489728

28. Ward LM, Idei A, Nakagawa M, Ueno Y, Fischer WW, McGlynn SE. Thermophilic Lithotrophy and Phototrophy in an Intertidal, Iron-rich, Geothermal Spring. bioRxiv [Internet]. 2018 Jan 1;428698. Available from: http://biorxiv.org/content/early/2018/09/27/428698.abstract

29. Langmead B, Salzberg SL. Fast gapped-read alignment with Bowtie 2. Nat Methods [Internet]. 2012 Apr 4 [cited 2018 May 1];9(4):357–9. Available from: http://www.ncbi.nlm.nih.gov/pubmed/22388286

30. Bray NL, Pimentel H, Melsted P, Pachter L. Near-optimal probabilistic RNA-seq quantification. Nat Biotechnol [Internet]. 2016 May 4 [cited 2018 May 2];34(5):525–7. Available from: http://www.nature.com/articles/nbt.3519

**Supplemental Figure Captions**

Supplemental Figure 1: Collection of seafloor images from R/V SuBastian for (a) Snail Pile vent at Illium, (b) Marker 131 vent at Alice Springs, (c) Snail Pit vent at Burke, (d) Voodoo Crater 1 at Hafa Adai, (e) Voodoo Crater 2 at Hafa Adai, (f) Alba vent at Hafa Adai, (g) Leaning Tower vent at Perseverance, and (h) Limpet Canyon vent at Perseverance.

Supplemental Figure 2: Relative abundance of 16S rRNA gene sequence 97% OTUs grouped by taxonomy for archaea primer set for all vent sites.

Supplemental Figure 3: Concatonated marker gene tree of Aquificae MAGs from this study contextualized with environmental MAGs and cultured genomes. Node diamonds are sized to bootstrap support, where Alphaproteobacteria root supports are 100 for reference.

Supplemental Figure 4: Concatonated marker gene tree of Epsilonbactaerota MAGs from this study contextualized with environmental MAGs and cultured genomes. Node diamonds are sized to bootstrap support, where Alphaproteobacteria root supports are 100 for reference. Star indicates “active” *Sulfurovum* MAGs with high recruitment from transcriptomes.

Supplemental Figure 5: Concatonated marker gene tree of Gammaproteobacteria MAGs from this study contextualized with environmental MAGs and cultured genomes. Node diamonds are sized to bootstrap support, where Alphaproteobacteria root supports are 100 for reference.

Supplemental Figure 6: (a) Cell abundance for all replicates of the Hafa Adai vent field Voodoo Crater-2 SIP-NanoSIMS incubations with temperature, hours of incubation, label added, cell abundance (cells/ml), and 95% confidence interval of cell abundance. (b) Nanoscale secondary ion mass spectrometry ion images from 9hr 13C-acetate incubations. 14N12C ion image (top) shows location of biomass, 1H2 ion image shows non-substrate activity (middle), and 13C ion image shows substrate specific activity (bottom). While scale bar is 1μm.

Supplemental Figure 7: Single-cell generation times calculated based on NanoSIMS data for ^2^H incorporation via ^2^H_2_O and ^13^C incorporation via ^13^C-acetate for 9 hr and 18 hr incubations at 80^o^C assuming 30 μM and 0 μM in situ acetate concentrations. Diagonal 1:1 line plotted for comparison.
