# Supplementary figures and images for: Active subseafloor microbial communities from Mariana back-arc venting fluids share metabolic strategies across different thermal niches and taxa
Source: ISME J. 2019 May 9;13(9):2264–79. doi: 10.1038/s41396-019-0431-y (PMC6775965; doi:10.1038/s41396-019-0431-y)

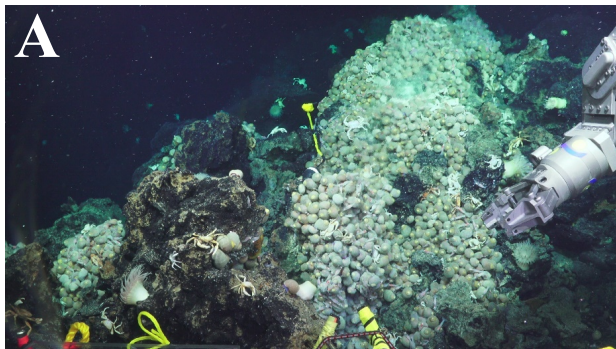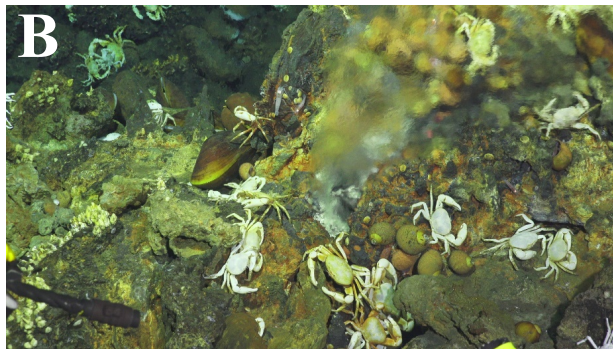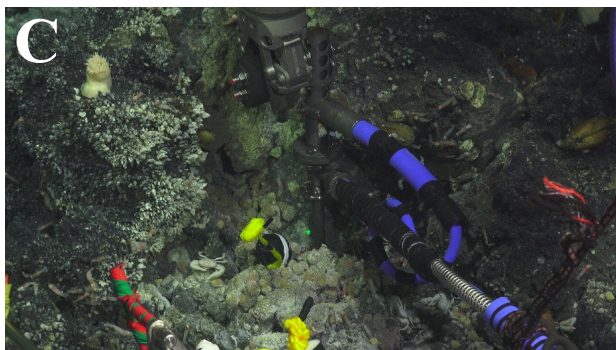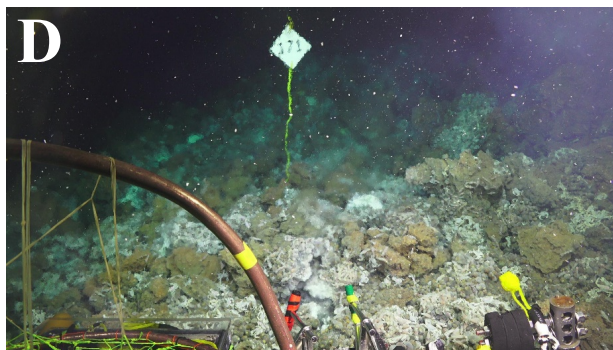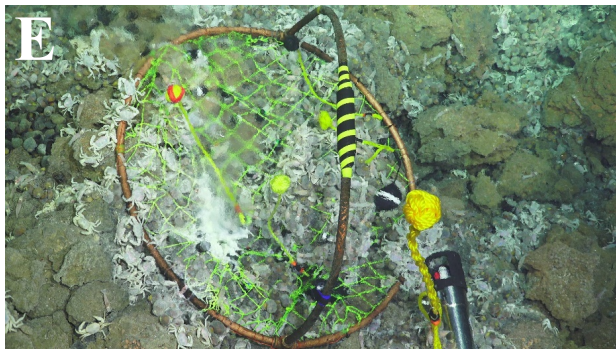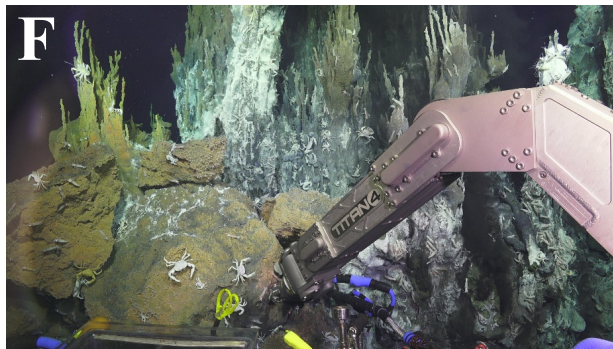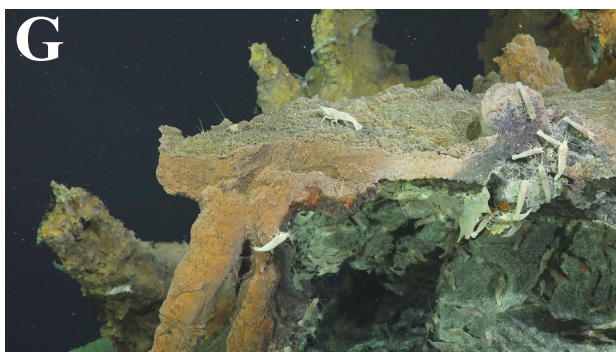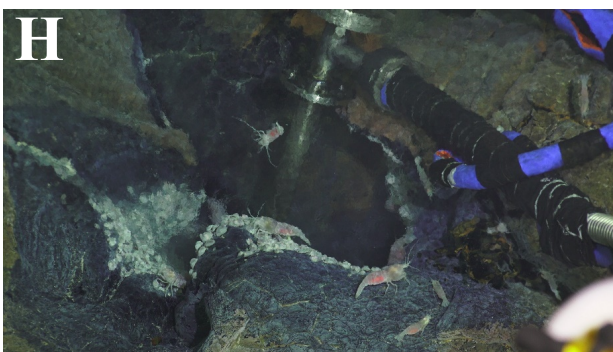

Supplement: Supplementary file 2 — Supplemental Figure 1 [file 41396_2019_431_MOESM2_ESM.pdf]

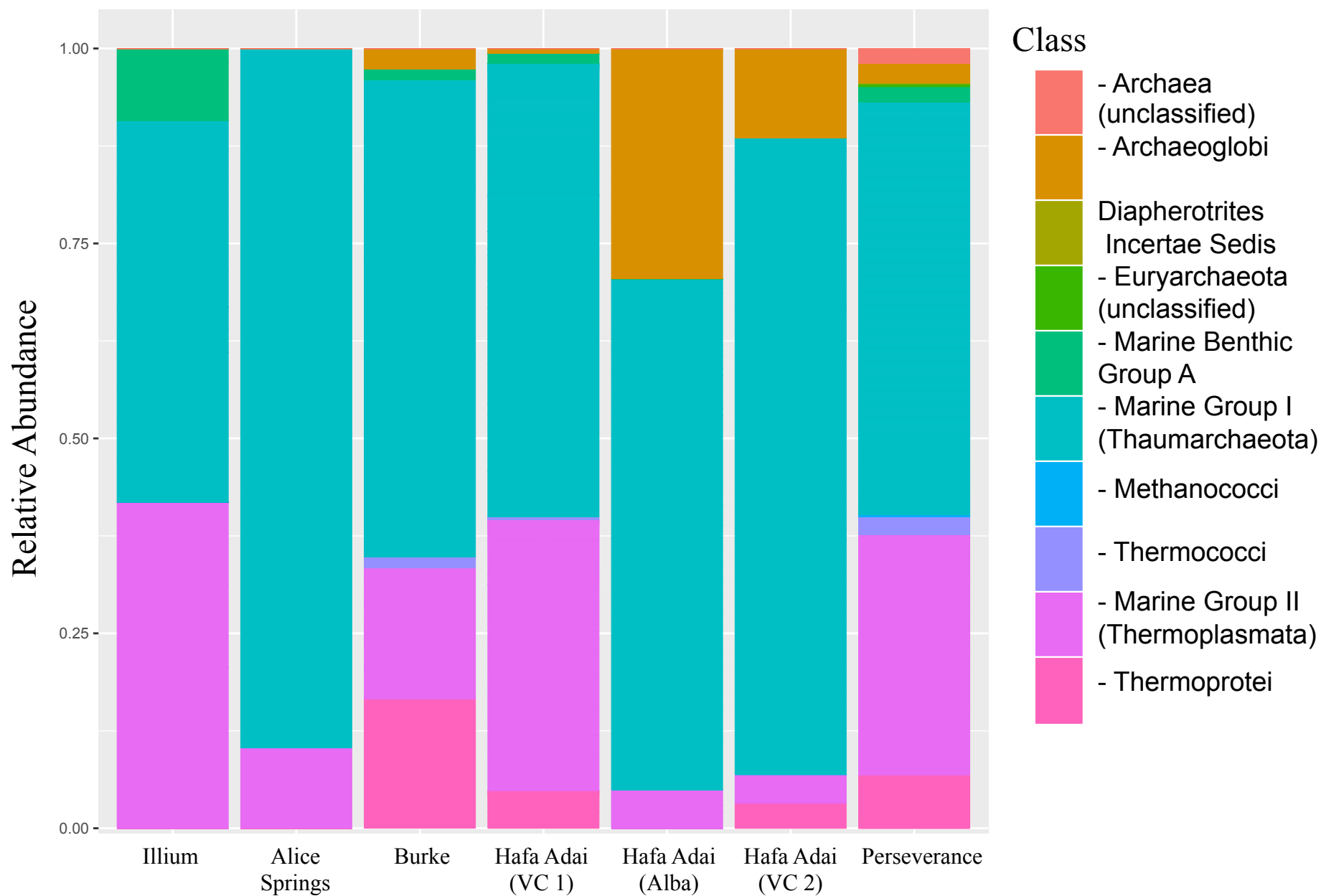

Supplement: Supplementary file 3 — Supplemental Figure 2 [file 41396_2019_431_MOESM3_ESM.pdf]

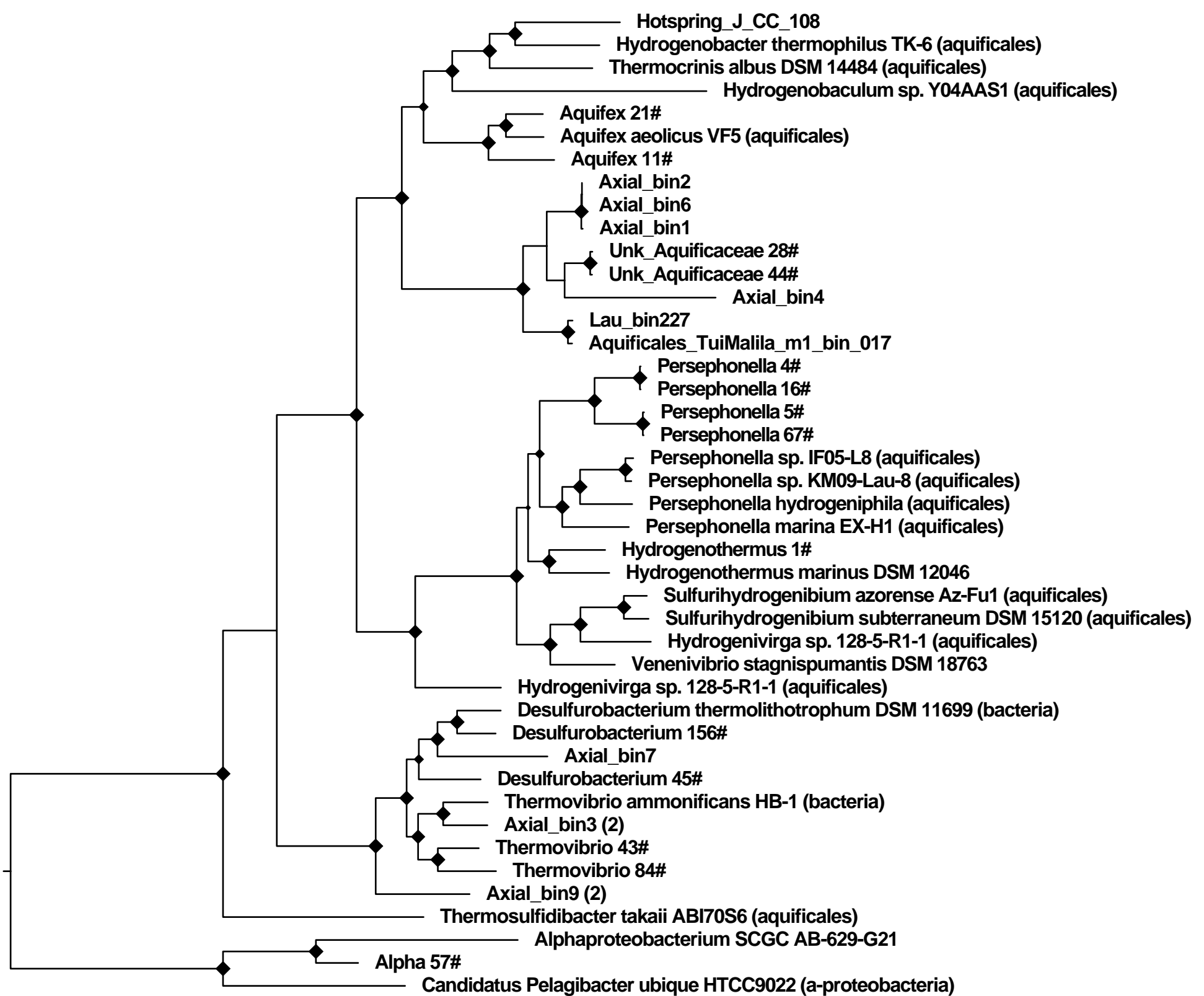

0.2

Supplement: Supplementary file 4 — Supplemental Figure 3 [file 41396_2019_431_MOESM4_ESM.pdf]

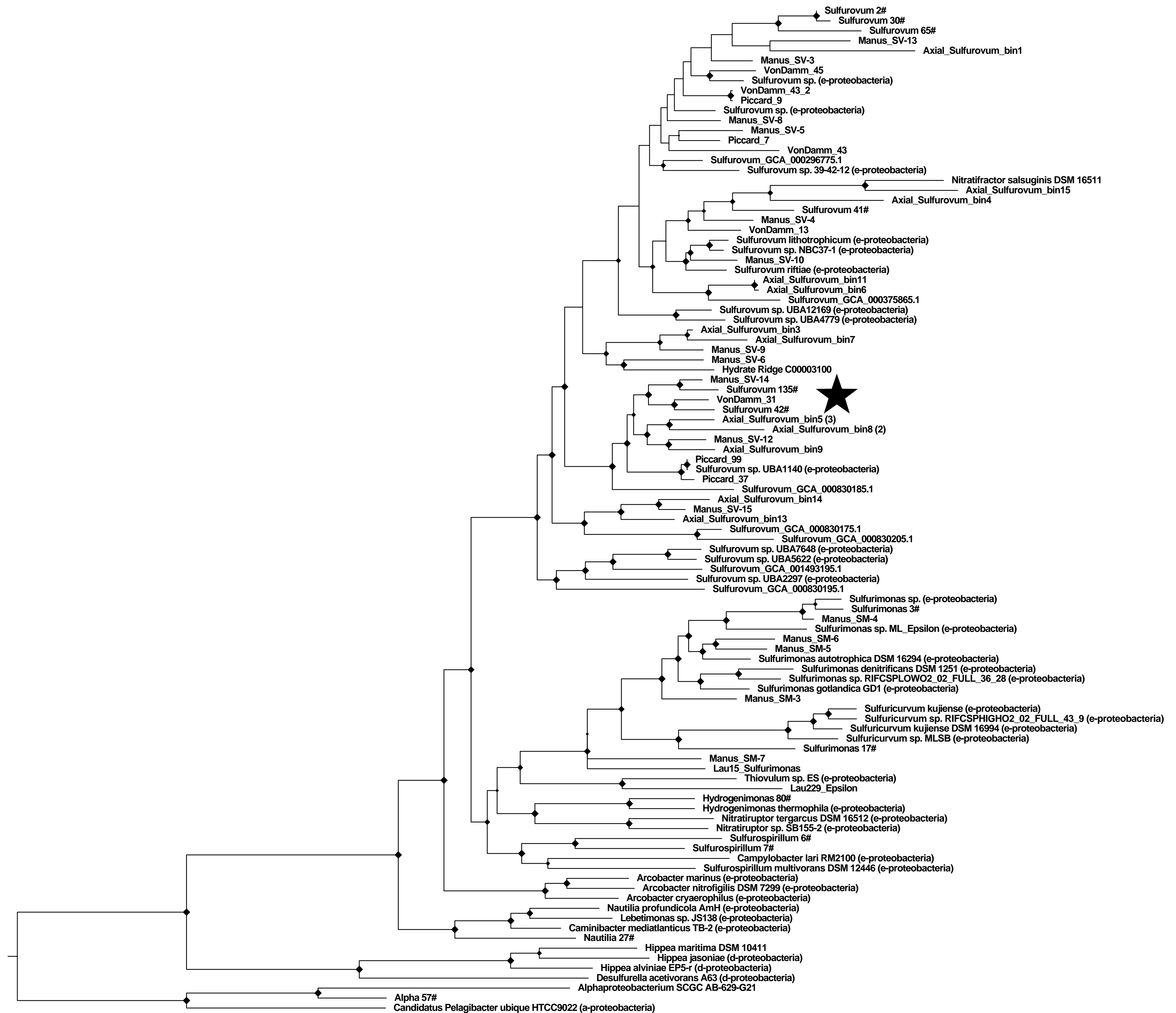

Supplement: Supplementary file 5 — Supplemental Figure 4 [file 41396_2019_431_MOESM5_ESM.pdf]

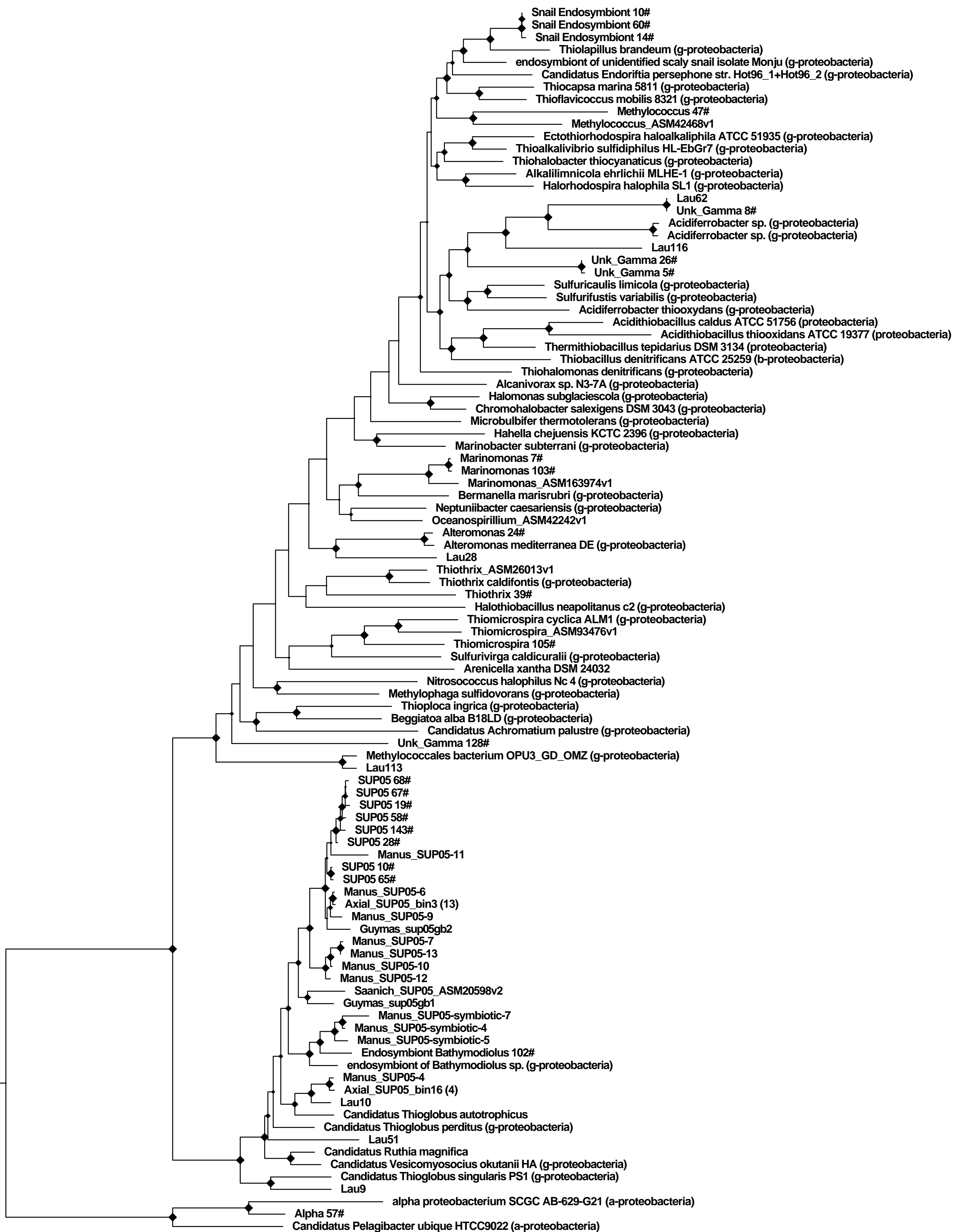

Supplement: Supplementary file 6 — Supplemental Figure 5 [file 41396_2019_431_MOESM6_ESM.pdf]

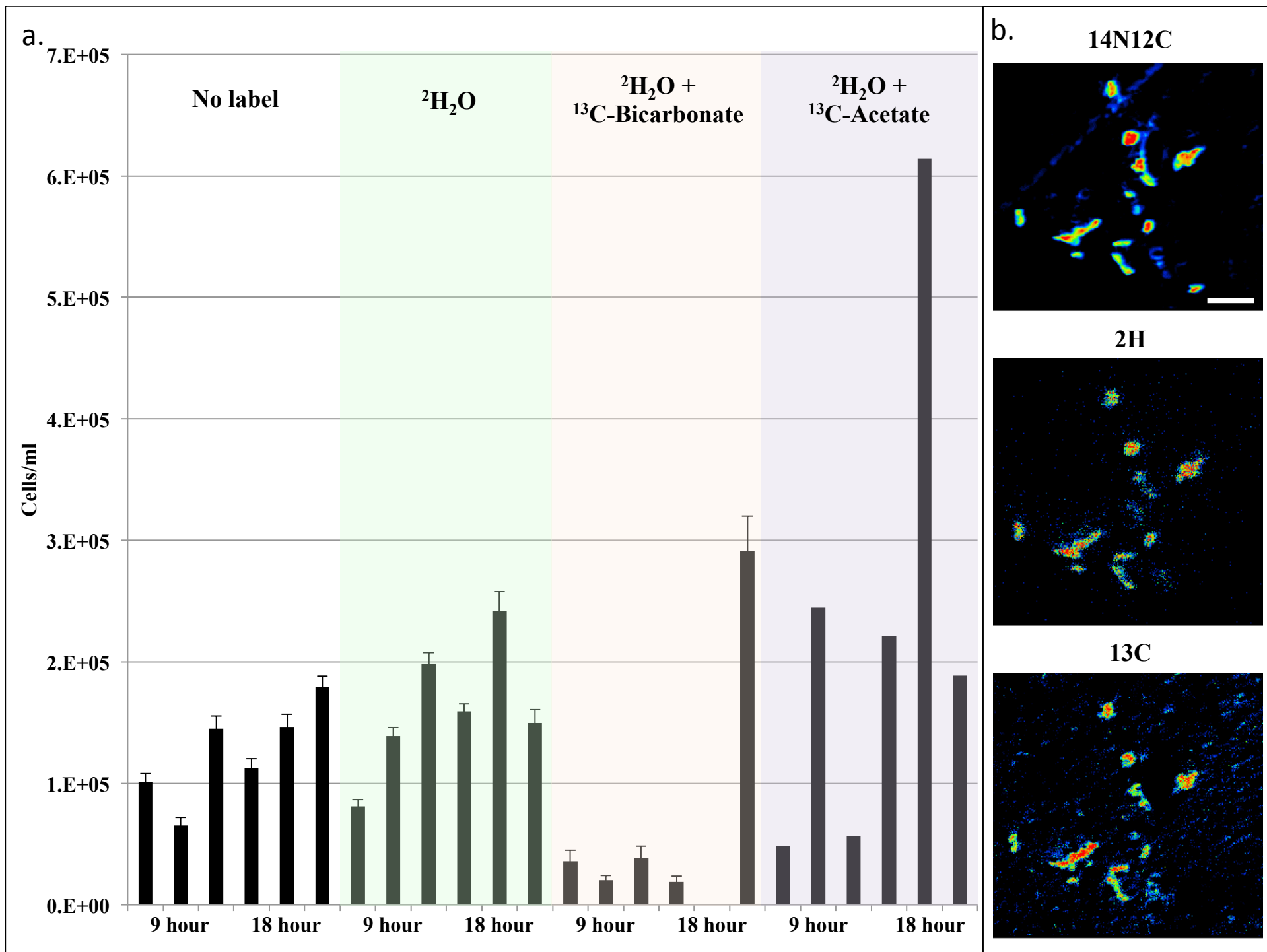

Supplement: Supplementary file 7 — Supplemental Figure 6 [file 41396_2019_431_MOESM7_ESM.pdf]

○  $^2\text{H}_2\text{O}$  and  $^{13}\text{C}$ -Acetate  
9 hr, 80°C

○  $^2\text{H}_2\text{O}$  and  $^{13}\text{C}$ -Acetate  
18 hr, 80°C

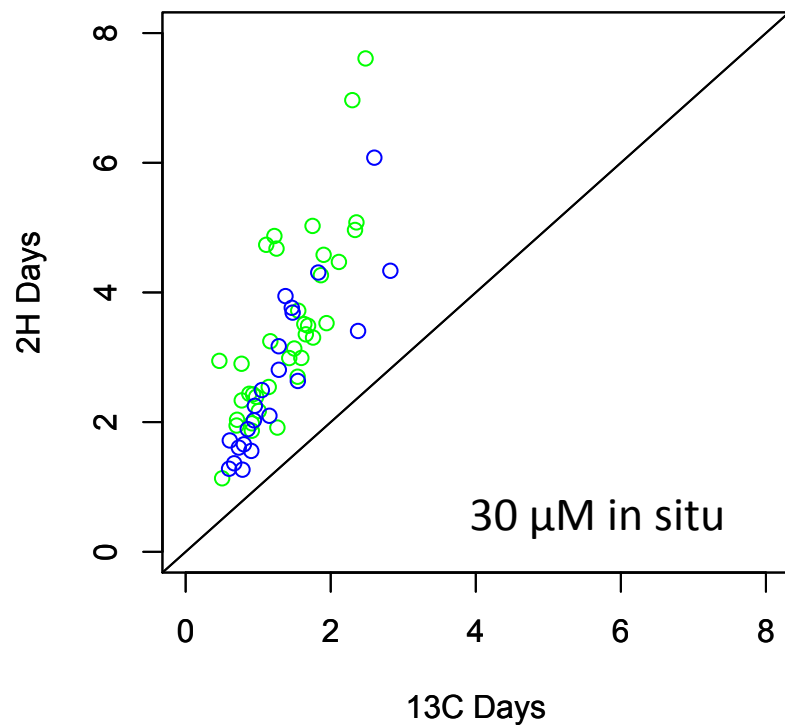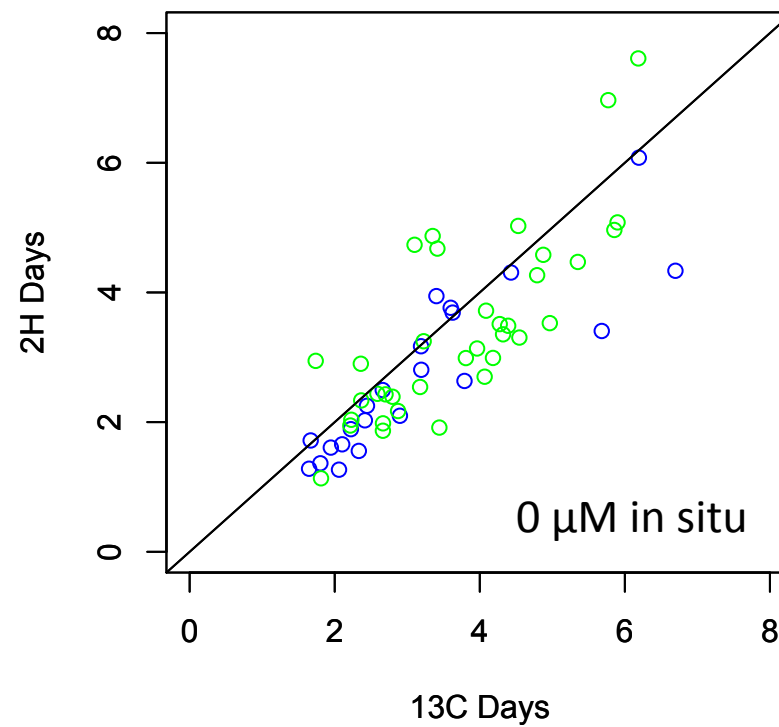

Supplement: Supplementary file 8 — Supplemental Figure 7 [file 41396_2019_431_MOESM8_ESM.pdf]
