# Supplementary material for: Active subseafloor microbial communities from Mariana back-arc venting fluids share metabolic strategies across different thermal niches and taxa
Source: ISME J. 2019 May 9;13(9):2264–79. doi: 10.1038/s41396-019-0431-y (PMC6775965; doi:10.1038/s41396-019-0431-y)
Supplement: Supplementary file 14 — Supplemental Table 6 [file 41396_2019_431_MOESM14_ESM.pdf]

Supplemental Table 6: All MAGs completion and contamination statistics, sorted by general taxonomic group and taxonomic classification based on Phylosift.

Group	Tree_classification	Completeness	Contamination	Heterogeneity	Corrected Contamination	Total Length	No. of Contigs	N50	Accession
Alpha	Deep Sea Vent Alpha 149	51.71	1.31	50	0.655	2.27E+06	298	9225	SAMN09407827
Alpha	Deep Sea Vent Alpha 23	58.45	2.41	100	0	8.30E+05	196	4382	SAMN09407828
Alpha	Deep Sea Vent Alpha 57	58.17	2.41	50	1.205	6.63E+05	149	4476	SAMN09407829
Alpha	Deep Sea Vent Alpha 62	53.04	2.41	0	2.41	5.68E+05	127	4677	SAMN09407830
Aquificales	Aquifex 11	87.99	1.3	42.86	0.74282	1.68E+06	218	10452	SAMN09407819
Aquificales	Aquifex 21	52.82	1.58	66.67	0.526614	1.69E+06	283	6810	SAMN09407820
Aquificales	Desulfurobacterium 156	55.01	2.25	66.67	0.749925	1.12E+06	88	17143	SAMN09407836
Aquificales	Desulfurobacterium 45	68.92	2.53	33.33	1.686751	1.17E+06	183	7513	SAMN09407837
Aquificales	Hydrogenothermus 1	93.29	0.41	0	0.41	1.49E+06	188	9211	SAMN09407844
Aquificales	Persephonella 16	70.71	0	0	0	2.09E+06	315	7857	SAMN09407857
Aquificales	Persephonella 4	72.8	0.42	0	0.42	1.03E+06	183	6297	SAMN09407858
Aquificales	Persephonella 5	68.26	0	0	0	9.43E+05	67	15907	SAMN09407859
Aquificales	Persephonella 67	82.42	0.81	50	0.405	1.16E+06	74	19899	SAMN09407860
Aquificales	Thermovibrio 43	63.16	2.63	0	2.63	1.08E+06	117	11596	SAMN09407894
Aquificales	Thermovibrio 84	55.27	0.42	0	0.42	7.76E+05	16	55537	SAMN09407895
Aquificales	Unk_Aquificaceae 28	77.77	2.85	100	0	1.03E+06	204	5546	SAMN09407899
Aquificales	Unk_Aquificaceae 44	86.09	1.53	50	0.765	7.08E+05	155	4826	SAMN09407900
Archaea	Archaeoglobus 85	71.02	0	0	0	1.20E+06	181	7357	SAMN09407821
Archaea	Hyperthermus 130	63.52	1.27	0	1.27	9.70E+05	50	26911	SAMN09407845
Archaea	MG II 35	50.54	0	0	0	1.05E+06	190	6117	SAMN09407851
Archaea	MG II 40	49.04	6.54	0	6.54	2.29E+06	329	8437	SAMN09407852
Archaea	Thaumarchaeota 147	54.13	6.8	85.71	0.97172	7.22E+05	144	5160	SAMN09407891
Delta	Desulfobulbus 32	63.85	2.68	40	1.608	1.78E+06	223	9765	SAMN09407831
Delta	Desulfobulbus 38	73.46	1.42	25	1.065	1.90E+06	368	5481	SAMN09407832
Delta	Desulfobulbus 51	51.45	1.55	40	0.93	1.83E+06	201	12347	SAMN09407833
Delta	Desulfobulbus 6_1	71.77	0.6	100	0	1.91E+06	236	9622	SAMN09407834
Delta	Desulfocapsa 30	66.14	6.63	5	6.2985	2.12E+06	441	5102	SAMN09407835
Delta	NaphS2 3_2	64.88	1.29	100	0	2.24E+06	327	7982	SAMN09407853
Delta	NaphS2 48	86.13	1.61	0	1.61	1.32E+06	309	4393	SAMN09407854
Delta	NaphS2 88	52.7	2.1	50	1.05	1.48E+06	259	6408	SAMN09407855
Delta	SAR324 151	86.64	1.26	100	0	1.41E+06	197	8968	SAMN09407862
Delta	SAR324 24	84.26	1.75	66.67	0.583275	6.36E+05	129	5152	SAMN09407863
Delta	SAR324 54	64.54	0.84	0	0.84	1.46E+06	332	4414	SAMN09407864
Delta	SAR324 58	80.99	0.91	50	0.455	2.12E+06	364	6571	SAMN09407865
Delta	SAR324 63_1	86.22	2.61	100	0	2.45E+06	315	9972	SAMN09407866
Delta	SAR324 63_2	65.62	0.47	50	0.235	9.59E+05	168	6321	SAMN09407867
Delta	Thermodesulfatator 87	57.58	0.89	50	0.445	1.28E+06	231	6053	SAMN09407892
Delta	Thermodesulfatator 95	81.31	0.93	33.33	0.620031	1.39E+06	140	13517	SAMN09407893
Epsilon	Hydrogenimonas 80	67.55	0	0	0	1.61E+06	94	21912	SAMN09407843
Epsilon	Nautilia 27	53.27	16.67	44.9	9.18517	6.90E+05	148	4818	SAMN09407856
Epsilon	Sulfurimonas 17	87.21	0.06	100	0	1.66E+06	246	8111	SAMN09407872
Epsilon	Sulfurimonas 3	85.21	1.36	75	0.34	1.43E+06	74	35788	SAMN09407873
Epsilon	Sulfurospirillum 6	83.1	3.91	43.75	2.199375	2.30E+06	232	13951	SAMN09407874
Epsilon	Sulfurospirillum 7	54.34	0.77	50	0.385	2.07E+06	383	5590	SAMN09407875
Epsilon	Sulfurovum 135	76.21	0	0	0	1.64E+06	173	12720	SAMN09407876
Epsilon	Sulfurovum 2	85.33	1.43	100	0	1.58E+06	228	8546	SAMN09407877
Epsilon	Sulfurovum 30	51.13	2.38	63.64	0.865368	1.09E+06	192	6359	SAMN09407878
Epsilon	Sulfurovum 41	61.07	2.77	87.5	0.34625	2.15E+06	330	7659	SAMN09407879
Epsilon	Sulfurovum 42	50	6.03	75	1.5075	1.20E+06	239	5267	SAMN09407880
Epsilon	Sulfurovum 65	65.39	3.17	6.67	2.958561	9.00E+05	101	11911	SAMN09407881
Gamma	Alteromonas 24	68.95	0.21	0	0.21	2.77E+06	145	28632	SAMN09407818
Gamma	Endosymbiont Bathymodiolus 102	79.09	1.99	75	0.4975	1.52E+06	180	14506	SAMN09407838
Gamma	Marinomonas 103	90.17	3.85	73.68	1.01332	3.49E+06	472	8890	SAMN09407848
Gamma	Marinomonas 7	53.82	1.07	0	1.07	1.88E+06	427	4557	SAMN09407849
Gamma	Methylococcus 47	88.59	1.32	12.5	1.155	1.98E+06	87	31277	SAMN09407850
Gamma	Snail Endosymbiont 10	59.73	1.13	50	0.565	3.54E+06	593	6988	SAMN09407869
Gamma	Snail Endosymbiont 14	64.23	0.9	0	0.9	1.50E+06	341	4490	SAMN09407870
Gamma	Snail Endosymbiont 60	94.54	1.22	0	1.22	1.29E+06	255	5241	SAMN09407871
Gamma	SUP05 10	68.91	3.19	62.5	1.19625	8.92E+05	162	6527	SAMN09407882
Gamma	SUP05 143	81.75	34.55	83.78	5.60401	1.40E+06	271	5642	SAMN09407883
Gamma	SUP05 19	92.41	1.18	0	1.18	8.03E+05	195	4039	SAMN09407884
Gamma	SUP05 28	87.42	47.92	85.71	6.847768	2.35E+06	295	9814	SAMN09407885
Gamma	SUP05 58	75.46	11.09	79.31	2.294521	1.04E+06	209	5348	SAMN09407886
Gamma	SUP05 65	70.81	8.26	76.47	1.943578	9.17E+05	162	6282	SAMN09407887
Gamma	SUP05 67	60.21	0.7	50	0.35	7.65E+05	154	5555	SAMN09407888
Gamma	SUP05 68	88.47	15.16	100	0	1.88E+06	369	5220	SAMN09407889
Gamma	Thiomicrospira 105	90.62	0.61	100	0	2.05E+06	40	75072	SAMN09407896
Gamma	Unk_Gamma 128	59.51	3.15	61.11	1.225035	2.14E+06	299	8301	SAMN09407897
Gamma	Thiothrix 39	68.97	0.86	0	0.86	1.02E+06	203	5533	SAMN09407898
Gamma	Unk_Gamma 26	53.86	2.03	62.5	0.76125	1.78E+06	164	13044	SAMN09407901
Gamma	Unk_Gamma 5	57.11	1.37	25	1.0275	1.24E+06	235	5812	SAMN09407902
Gamma	Unk_Gamma 8	77.44	2.44	0	2.44	2.81E+06	127	35848	SAMN09407903
Other Bacteria	Actinobacteria 38	50	1.72	0	1.72	1.56E+06	170	11525	SAMN09407816
Other Bacteria	Actinobacteria 79	68.28	0.51	0	0.51	1.96E+06	51	60194	SAMN09407817
Other Bacteria	Bacteroidetes 44	56.9	0	0	0	3.27E+06	113	48000	SAMN09407822
Other Bacteria	Bacteroidetes 90	88.91	2.19	0	2.19	1.11E+06	97	17226	SAMN09407823
Other Bacteria	Chloroflexi 5	92.24	1.98	50	0.99	3.37E+06	118	54542	SAMN09407824
Other Bacteria	Chloroflexi 58	71.85	9.65	22.22	7.50577	2.74E+06	527	5679	SAMN09407825
Other Bacteria	Clostridia 154	50.06	0.64	50	0.32	8.76E+05	154	6572	SAMN09407826
Other Bacteria	Firmicutes 7	94.02	1.08	0	1.08	2.98E+06	108	52127	SAMN09407839
Other Bacteria	Flavobacteria 22	72.17	2.94	25	2.205	1.35E+06	229	6772	SAMN09407840
Other Bacteria	Flavobacteria 92	85.14	6.91	60.47	2.731523	3.68E+06	457	9821	SAMN09407841
Other Bacteria	Fusobacteria 22	67.98	1.12	0	1.12	1.03E+06	216	5123	SAMN09407842
Other Bacteria	Marinimicrobia 48	51.2	1	66.67	0.3333	1.91E+06	379	5487	SAMN09407846
Other Bacteria	Marinimicrobia 63	51.75	1.81	100	0	9.07E+05	161	6302	SAMN09407847
Other Bacteria	Planctomycetes 59	80.55	1.61	0	1.61	2.68E+06	248	14030	SAMN09407861
Other Bacteria	SAR406 55	54.51	5.04	0	5.04	1.03E+06	246	4159	SAMN09407868
Other Bacteria	Tenericutes 1_4	60.91	5.61	65	1.9635	4.06E+05	71	6248	SAMN09407890
Other Bacteria	Verrucomicrobia 15	72.27	3.89	62.5	1.45875	2.77E+06	319	11118	SAMN09407904
