# Supplementary material for: Active subseafloor microbial communities from Mariana back-arc venting fluids share metabolic strategies across different thermal niches and taxa
Source: ISME J. 2019 May 9;13(9):2264–79. doi: 10.1038/s41396-019-0431-y (PMC6775965; doi:10.1038/s41396-019-0431-y)
Supplement: Supplementary file 15 — Supplemental Table 8 [file 41396_2019_431_MOESM15_ESM.pdf]

		Voodoo Crater-1	Voodoo Crater-2	Alba	None_18hr	D2O_9hr	DIC_9hr	DIC_18hr	Ac_9hr	Ac_18hr
Vent Specific OTUs	Sulfurovum_OTU5	20.531%	34.294%	21.857%	0.249%	1.198%	2.346%	7.179%	3.390%	0.532%
	Desulfurobacterium_OTU2	0.178%	0.154%	0.303%	80.660%	11.902%	0.003%	0.001%	2.043%	14.632%
	Desulfurobacterium_OTU1	0.007%	0.000%	0.016%	9.850%	18.904%	0.000%	0.000%	23.209%	29.006%
	Desulfurobacterium_OTU52	0.004%	0.001%	0.002%	1.347%	0.293%	0.000%	0.000%	0.030%	0.271%
	Aquifex_OTU62	0.000%	0.000%	0.000%	0.002%	0.000%	0.000%	0.000%	0.000%	1.166%
	Pseudoalteromonas_OTU6	0.769%	0.014%	0.053%	0.092%	0.095%	0.350%	10.992%	0.117%	0.032%
Sequencing Contaminant OTUs	Actinobacteria	0.000%	0.000%	0.000%	1.715%	21.798%	15.307%	28.692%	16.584%	12.908%
	Alphaproteobacteria	0.000%	0.000%	0.000%	0.024%	1.202%	19.204%	2.977%	1.517%	1.846%
	Bacilli	0.000%	0.000%	0.000%	0.138%	0.712%	1.366%	0.056%	1.376%	0.417%
	Bacteroidia	0.000%	0.000%	0.000%	1.087%	17.279%	15.885%	14.589%	13.602%	4.784%
	Betaproteobacteriales	0.001%	0.000%	0.000%	0.300%	1.336%	13.111%	1.909%	1.684%	1.799%
	Gammaproteobacteria	0.000%	0.009%	0.000%	0.454%	3.411%	21.411%	7.668%	6.894%	7.532%
