# Supplementary material for: Active subseafloor microbial communities from Mariana back-arc venting fluids share metabolic strategies across different thermal niches and taxa
Source: ISME J. 2019 May 9;13(9):2264–79. doi: 10.1038/s41396-019-0431-y (PMC6775965; doi:10.1038/s41396-019-0431-y)
Supplement: Supplementary file 16 — Supplemental Table 7 [file 41396_2019_431_MOESM16_ESM.pdf]

Supplemental Table 7: Percent recruitment of reads to each MAG from all metagenomes, metatranscriptomes, and RNA-SIP metatranscriptomes.

		Ilium		Alice Springs		Burke			Hafa Adai (VC-1)		Hafa Adai (Alba)			Hafa Adai (VC-2)					Perseverence (LC)		Perseverence Plume	
Group	Tree_classification	MetaG	SIP	MetaG	MetaT	MetaG	SIP	MetaT	MetaG	MetaT	MetaG	SIP	MetaT	MetaG	SIP	SIP_18_80	SIP_9_80	MetaT	MetaG	MetaT	MetaG	MetaT
Alpha	Deep Sea Vent Alpha 149	0.1	0.0	0.2	0.1	0.0	0.0	0.0	0.1	0.1	0.0	0.0	0.0	0.0	0.0	0.0	0.0	0.0	0.0	0.0	0.2	0.3
Alpha	Deep Sea Vent Alpha 23	0.1	0.0	0.4	0.0	0.1	0.0	0.0	0.2	0.1	0.1	0.0	0.0	0.1	0.0	0.0	0.0	0.0	0.0	0.0	0.3	0.1
Alpha	Deep Sea Vent Alpha 57	0.2	0.0	0.3	0.5	0.1	0.0	0.0	0.2	0.1	0.1	0.0	0.1	0.1	0.0	0.0	0.0	0.0	0.0	0.0	0.4	0.1
Alpha	Deep Sea Vent Alpha 62	0.2	0.0	0.3	0.1	0.1	0.0	0.0	0.2	0.0	0.1	0.0	0.0	0.0	0.0	0.0	0.0	0.0	0.0	0.0	0.4	0.1
Aquificales	Aquifex 11	0.0	0.0	0.4	0.9	0.0	0.0	0.0	0.0	0.4	0.0	0.0	0.0	0.0	0.0	0.0	0.0	0.0	0.0	0.0	0.0	0.0
Aquificales	Aquifex 21	0.0	0.0	4.4	7.1	0.0	0.0	0.0	0.0	0.9	0.0	0.0	0.2	0.0	0.0	0.0	0.0	0.4	0.0	0.0	0.0	0.0
Aquificales	Desulfurobacterium 156	0.1	0.0	0.0	0.0	0.1	0.0	0.0	0.0	0.0	0.0	0.0	0.0	0.0	0.0	0.0	0.0	0.0	0.0	0.3	0.0	0.0
Aquificales	Desulfurobacterium 45	0.1	0.0	0.0	0.1	0.0	0.0	0.2	0.0	0.4	0.1	0.0	0.2	0.0	0.0	0.0	0.0	0.1	0.0	0.1	0.0	0.0
Aquificales	Hydrogenothermus 1	0.0	0.0	0.0	0.5	0.0	0.0	0.0	0.0	0.0	0.0	0.0	0.0	0.0	0.0	88.9	0.0	0.0	0.0	0.0	0.1	2.2
Aquificales	Persephonella 16	0.1	0.0	0.0	0.3	0.0	0.0	0.0	0.0	0.0	0.0	0.0	0.0	0.0	0.0	1.8	0.0	0.0	0.0	0.0	0.0	0.0
Aquificales	Persephonella 4	0.4	0.0	0.3	0.5	0.1	0.0	0.0	0.0	0.0	0.0	0.0	0.1	0.0	0.0	0.4	0.0	0.0	0.0	0.0	0.0	0.0
Aquificales	Persephonella 5	0.0	0.0	0.2	0.2	0.1	0.0	0.0	0.0	0.2	0.0	0.0	0.4	0.0	0.0	0.1	0.0	0.1	0.0	0.0	0.0	0.0
Aquificales	Persephonella 67	0.0	0.0	0.1	0.4	0.0	0.0	0.0	0.0	0.1	0.0	0.0	0.0	0.0	0.0	3.3	0.0	0.0	0.0	0.0	0.0	0.0
Aquificales	Thermovibrio 43	0.0	0.0	0.0	0.0	0.0	0.0	0.2	0.1	0.2	0.1	0.0	0.1	0.1	0.0	0.0	0.0	0.1	0.0	0.0	0.0	0.0
Aquificales	Thermovibrio 84	0.0	0.0	0.0	0.0	0.0	0.0	0.0	0.0	0.0	0.0	0.0	0.0	0.0	0.0	0.0	0.0	0.0	0.0	0.0	0.0	0.0
Aquificales	Unk_Aquificaceae 28	0.0	0.0	0.0	0.7	0.2	0.0	0.8	0.0	0.4	0.0	0.0	0.7	0.0	0.0	0.0	0.0	0.2	0.0	0.0	0.0	0.0
Aquificales	Unk_Aquificaceae 44	0.0	0.0	0.1	0.4	0.0	0.0	0.7	0.0	0.4	0.2	0.0	0.7	0.0	0.0	0.0	0.0	0.1	0.0	0.0	0.0	0.0
Archaea	Archaeoglobus 85	0.0	0.0	0.0	0.0	0.0	0.0	0.0	0.0	0.0	0.4	0.6	1.6	0.0	0.0	0.0	0.0	0.0	0.0	0.1	0.0	0.0
Archaea	Hyperthermus 130	0.0	0.0	0.0	0.0	0.0	0.0	0.5	0.0	0.3	0.0	0.0	0.0	0.0	0.0	0.0	0.0	0.0	0.0	0.0	0.0	0.0
Archaea	MG II 35	0.1	0.0	0.3	0.4	0.1	0.0	0.0	0.3	0.5	0.1	0.0	0.5	0.1	0.0	0.0	0.0	0.0	0.0	0.1	0.5	1.9
Archaea	MG II 40	0.1	0.0	0.1	1.7	0.0	0.0	0.0	0.1	0.4	0.1	0.0	0.0	0.0	0.0	0.0	0.0	0.2	0.0	0.0	0.4	1.4
Archaea	Thaumarchaeota 147	0.1	0.0	0.6	0.1	0.1	0.0	0.0	0.3	0.0	0.1	0.0	0.0	0.1	0.0	0.0	0.0	0.0	0.0	0.0	0.5	0.2
Delta	Desulfobulbus 32	0.0	0.0	0.0	0.0	0.0	0.0	0.0	0.2	0.1	0.2	0.0	0.1	0.6	0.0	0.0	0.0	0.2	0.0	0.2	0.0	0.0
Delta	Desulfobulbus 38	0.0	0.0	0.0	0.0	0.0	0.0	0.0	0.3	0.0	0.3	0.0	0.1	1.0	0.0	0.0	0.0	0.2	0.0	0.1	0.0	0.0
Delta	Desulfobulbus 51	0.1	0.0	0.0	0.0	0.0	0.0	0.0	0.0	0.0	0.0	0.0	0.1	0.1	0.0	0.0	0.0	0.1	0.0	0.1	0.0	0.0
Delta	Desulfobulbus 6_1	0.0	0.0	0.0	0.0	0.0	0.0	0.0	0.0	0.0	0.1	0.0	0.0	0.2	0.0	0.0	0.0	0.0	0.0	0.0	0.0	0.0
Delta	Desulfocapsa 30	0.0	0.0	0.0	0.1	0.0	0.0	0.0	0.0	0.0	0.0	0.0	0.0	0.0	0.0	0.0	0.0	0.0	0.5	1.3	0.0	0.0
Delta	NaphS2 3_2	0.1	0.0	0.0	0.0	0.0	0.0	0.0	0.0	0.0	0.0	0.0	0.0	0.1	0.0	0.0	0.0	0.0	0.0	0.2	0.0	0.0
Delta	NaphS2 48	0.1	0.0	0.0	0.0	0.0	0.0	0.0	0.0	0.0	0.0	0.0	0.0	0.1	0.0	0.0	0.0	0.1	0.0	0.0	0.0	0.0
Delta	NaphS2 88	0.0	0.0	0.0	0.0	0.1	0.0	0.0	0.0	0.1	0.0	0.0	0.0	0.0	0.0	0.0	0.0	0.0	0.0	0.0	0.0	0.0
Delta	SAR324 151	0.1	0.0	0.2	0.3	0.0	0.0	0.0	0.2	0.1	0.1	0.0	0.0	0.0	0.0	0.0	0.0	0.0	0.0	0.1	0.3	0.8
Delta	SAR324 24	0.3	0.0	0.7	0.2	0.2	0.0	0.0	0.6	0.4	0.2	0.0	0.1	0.1	0.0	0.0	0.0	0.2	0.0	0.3	0.7	0.8
Delta	SAR324 54	0.1	0.0	0.2	0.4	0.0	0.0	0.0	0.2	0.2	0.1	0.0	0.0	0.0	0.0	0.0	0.0	0.0	0.0	0.1	0.3	1.7
Delta	SAR324 58	0.5	0.0	0.6	2.2	0.2	0.0	0.0	0.7	0.5	0.3	0.0	0.1	0.2	0.0	0.0	0.0	0.5	0.1	0.1	1.0	1.1
Delta	SAR324 63	0.4	0.0	0.6	0.3	0.2	0.0	0.0	0.6	0.2	0.2	0.0	0.0	0.1	0.0	0.0	0.0	0.0	0.0	0.1	0.7	1.0
Delta	SAR324 63	0.4	0.0	0.8	0.3	0.0	0.0	0.0	0.6	0.1	0.3	0.0	0.0	0.1	0.0	0.0	0.0	0.0	0.0	0.0	0.9	1.2
Delta	Thermodesulfatator 87	0.0	0.0	0.0	0.0	0.0	0.0	0.0	0.0	0.0	0.0	0.0	0.0	0.0	0.0	0.0	0.0	0.0	0.0	0.0	0.0	0.0
Delta	Thermodesulfatator 95	0.0	0.0	0.0	0.0	0.0	0.0	0.0	0.0	0.0	0.0	0.0	0.0	0.0	0.0	0.0	0.0	0.0	0.0	0.0	0.0	0.0
Epsilon	Hydrogenimonas 80	0.0	10.3	0.0	0.0	0.0	6.2	0.0	0.0	0.0	0.0	0.1	0.0	0.0	52.4	0.0	100.0	0.0	0.0	0.3	0.1	0.4
Epsilon	Nautilia 27	0.2	0.0	0.0	0.0	0.0	0.0	0.1	0.1	0.9	0.2	0.0	0.9	0.1	0.0	0.0	0.0	0.2	0.0	0.0	0.0	0.0
Epsilon	Sulfurimonas 17	0.0	0.0	0.1	0.0	0.0	0.0	0.0	0.2	0.0	0.1	0.0	0.0	0.0	0.0	0.0	0.0	0.0	0.0	0.0	0.0	0.0
Epsilon	Sulfurimonas 3	0.1	0.0	0.0	0.4	0.0	0.0	0.0	0.1	0.2	0.0	0.0	0.1	0.0	0.0	0.0	0.0	0.1	0.0	0.3	0.1	0.5
Epsilon	Sulfurospirillum 6	0.1	0.0	0.0	0.0	0.0	0.0	0.0	0.0	0.0	0.0	0.0	0.1	0.0	0.0	0.0	0.0	0.0	0.0	0.0	0.0	0.0
Epsilon	Sulfurospirillum 7	0.1	0.0	0.0	0.0	0.0	0.0	0.0	0.0	0.0	0.0	0.0	0.0	0.0	0.0	0.0	0.0	0.0	0.0	0.0	0.0	0.0
Epsilon	Sulfurovum 135	0.1	0.0	0.0	0.0	0.1	0.0	0.1	0.2	2.6	0.4	0.0	3.2	0.2	0.0	0.0	0.0	1.7	0.1	3.1	0.0	0.2
Epsilon	Sulfurovum 2	0.2	0.0	0.0	0.0	0.0	0.0	0.0	0.6	0.1	0.3	0.0	0.0	0.4	0.0	0.0	0.0	0.1	0.0	0.0	0.0	0.0
Epsilon	Sulfurovum 30	0.2	0.0	0.0	0.0	0.1	0.0	0.0	0.5	0.1	0.2	0.0	0.0	0.3	0.0	0.0	0.0	0.0	0.0	0.1	0.0	0.0
Epsilon	Sulfurovum 41	0.1	0.0	0.0	0.1	0.0	0.0	0.0	0.1	0.1	0.0	0.0	0.0	0.0	0.0	0.0	0.0	0.0	0.0	0.0	0.0	0.0
Epsilon	Sulfurovum 42	0.1	0.0	0.0	0.0	0.1	0.0	0.0	1.0	1.1	1.6	0.0	2.2	1.4	0.0	0.0	0.0	1.0	0.8	2.8	0.0	0.1
Epsilon	Sulfurovum 65	0.1	0.0	0.0	0.0	0.0	0.0	0.0	0.2	0.2	0.1	0.0	0.1	0.2	0.0	0.0	0.0	0.1	0.0	0.0	0.0	0.0
Gamma	Alteromonas 24	1.8	0.0	0.0	0.1	0.0	0.0	0.0	0.0	0.0	0.2	0.0	1.1	0.0	0.0	0.0	0.0	0.0	0.0	0.1	0.0	0.0
Gamma	Endosymbiont Bathymodiolus 102	0.0	0.0	0.0	0.0	0.1	0.0	0.0	0.0	0.0	0.0	0.0	0.0	0.0	0.0	0.0	0.0	0.0	0.0	0.0	0.0	0.0
Gamma	Marinomonas 103	2.5	0.0	0.1	2.1	0.0	0.0	0.0	0.0	0.1	0.0	0.0	0.2	0.0	0.0	0.0	0.0	0.0	0.1	1.0	0.0	0.0
Gamma	Marinomonas 7	6.9	0.0	0.5	1.2	0.1	0.0	0.0	0.0	0.3	0.0	0.0	1.5	0.0	0.0	0.0	0.0	0.0	0.3	4.5	0.0	0.0
Gamma	Methylococcus 47	0.0	0.0	0.1	0.6	0.0	0.0	0.0	0.0	0.1	0.0	0.0	0.0	0.0	0.0	0.0	0.0	0.0	0.0	0.0	0.1	0.8
Gamma	Snail Endosymbiont 10	0.1	0.0	0.3	1.0	0.0	0.0	0.0	0.3	0.1	0.1	0.0	0.1	0.1	0.0	0.0	0.0	0.1	0.0	0.5	1.7	13.8
Gamma	Snail Endosymbiont 14	1.9	0.0	0.1	0.1	0.1	0.0	0.0	0.3	0.2	0.1	0.0	0.3	0.6	0.0	0.0	0.0	2.3	0.0	0.0	0.0	0.0
Gamma	Snail Endosymbiont 60	0.5	0.0	0.0	0.1	0.0	0.0	0.0	0.1	0.1	0.0	0.0	0.3	0.1	0.0	0.0	0.0	2.0	0.0	0.0	0.0	0.0
Gamma	SUP05 10	0.1	0.0	0.2	0.1	0.1	0.0	0.0	0.2	0.0	0.2	0.0	0.2	0.3	0.0	0.0	0.0	0.0	0.0	0.1	0.9	3.0
Gamma	SUP05 143	0.1	0.0	0.3	0.2	0.1	0.0	0.0	0.3	0.2	0.1	0.0	0.2	0.1	0.0	0.0	0.0	0.2	0.0	0.4	2.2	17.4
Gamma	SUP05 19	0.0	0.0	0.0	0.0	0.0	0.0	0.0	0.0	0.0	0.0	0.0	0.0	0.0	0.0	0.0	0.0	0.0	0.0	0.0	0.0	0.0
Gamma	SUP05 28	0.3	0.0	0.9	0.1	0.3	0.0	0.0	1.0	0.5	0.5	0.0	0.9	0.4	0.0	0.0	0.0	0.8	0.1	2.1	4.3	12.8
Gamma	SUP05 58	0.1	0.0	0.3	0.2	0.1	0.0	0.0	0.3	0.1	0.1	0.0	0.2	0.1	0.0	0.0	0.0	0.2	0.0	0.3	2.0	18.0
Gamma	SUP05 65	0.1	0.0	0.2	0.0	0.1	0.0	0.0	0.2	0.0	0.1	0.0	0.1	0.1	0.0	0.0	0.0	0.0	0.0	0.1	0.9	3.0
Gamma	SUP05 67	0.2	0.0	0.4	0.1	0.1	0.0	0.0	0.7	0.1	0.3	0.0	0.1	0.1	0.0	0.0	0.0	0.0	0.1	0.2	3.0	10.2
Gamma	SUP05 68	0.2	0.0	0.7	0.2	0.0	0.0	0.0	0.8	0.2	0.4	0.0	0.2	0.2	0.0	0.0	0.0	0.1	0.1	0.4	4.0	18.2
Gamma	Thiomicrospira 105	0.8	0.0	0.0	0.1	0.0	0.0	0.0	0.0	0.0	0.0	0.0	0.0	0.0	0.0	0.0	0.0	0.0	0.0	0.4	0.0	0.0
Gamma	Thiothrix 128	0.0	0.0	0.0	0.0	0.3	0.0	0.1	0.0	0.0	0.0	0.0	0.0	0.0	0.0	0.0	0.0	0.0	0.0	0.0	0.0	0.0
Gamma	Thiothrix 39	0.1	0.0	0.0	0.0	0.0	0.0	0.0	0.0	0.1	0.0	0.0	0.0	0.0	0.0	0.0	0.0	0.0	0.0	0.0	0.0	0.0
Gamma	Unk_Gamma 26	0.2	0.0	0.0	0.0	0.0	0.0	0.0	0.1	0.0	0.0	0.0	0.0	0.0	0.0	0.0	0.0	0.0	0.0	0.0	0.0	0.0
Gamma	Unk_Gamma 5	0.7	0.0	0.0	0.0	0.0	0.0	0.0	0.2	0.0	0.0	0.0	0.0	0.1	0.0	0.0	0.0	0.0	0.0	0.0	0.0	0.0
Gamma	Unk_Gamma 8	0.1	0.0	0.1	0.4	0.0	0.0	0.0	0.1	0.1	0.0	0.0	0.0	0.0	0.0	0.0	0.0	0.0	0.0	0.0	0.3	0.5
Other Bacteria	Actinobacteria 38	0.1	0.0	0.1	0.1	0.0	0.0	0.0	0.1	0.0	0.0	0.0	0.1	0.0	0.0	0.0	0.0	0.0	0.0	0.0	0.2	0.1
Other Bacteria	Actinobacteria 79	0.0	0.0	0.0	0.0	0.0	0.0	0.0	0.0	0.0	0.0	0.0	0.1	0.0	0.0	0.0	0.0	0.0	0.0	0.0	0.3	0.0
Other Bacteria	Bacteroidetes 44	0.2	0.0	0.0	0.0	0.0	0.0	0.0	0.1	0.1	0.0	0.0	0.0	0.0	0.0	0.0	0.0	0.1	0.0	0.0	0.0	0.0
Other Bacteria	Bacteroidetes 90	0.0	0.0	0.0	0.0	0.0	0.0	0.0	0.0	0.0	0.3	0.0	0.2	0.0	0.0	0.0	0.0	0.0	0.0	0.0	0.0	0.0
Other Bacteria	Chloroflexi 5	0.1	0.0	0.1	1.2	0.0	0.0	0.0	0.2	0.3	0.1	0.0	0.2	0.0	0.0	0.0	0.0	0.1	0.0	0.1	0.4	1.9
Other Bacteria	Chloroflexi 58	0.3	0.0	0.5	0.5	0.1	0.0	0.0	0.4	0.2	0.1	0.0	0.1	0.0	0.0	0.0	0.0	0.0	0.0	0.1	1.0	3.6
Other Bacteria	Clostridia 154	0.1	0.0	0.0	0.0	0.0	0.0	0.0	0.0	0.0	0.0	0.0	0.0	0.0	0.0	0.0	0.0	0.0	0.0	0.0	0.0	0.0
Other Bacteria	Firmicutes 7	0.1	0.4	0.1	1.3	0.0	0.0	0.0	0.2	0.3	0.0	0.0	0.1	0.0	0.0	0.0	0.0	0.0	0.0	0.1	0.3	0.5
Other Bacteria	Flavobacteria 22	0.1	0.0	0.0	0.0	0.0	0.0	0.0	0.0	0.0	0.0	0.0	0.1	0.0	0.0	0.0	0.0	0.1	0.0	0.0	0.0	0.0
Other Bacteria	Flavobacteria 92	0.0	0.0	0.0	0.0	0.0	0.0	0.0	0.1	0.0	0.4	0.0	0.2	0.0	0.0	0.0	0.0	0.0	0.0	0.0	0.0	0.0
Other Bacteria	Fusobacteria 22	0.8	0.0	0.0	0.0	0.0	0.0	0.0	0.1	0.3	0.2	0.0	0.0	0.2	0.0	0.0	0.0	0.1	0.0	0.0	0.0	0.0
Other Bacteria	Marinimicrobia 48	0.1	0.0	0.2	0.0	0.0	0.0	0.0	0.1	0.0	0.1	0.0	0.0	0.1	0.0	0.0	0.0	0.0	0.0	0.0	0.3	0.5
Other Bacteria	Marinimicrobia 63	0.1	0.0	0.1	0.2	0.0	0.0	0.0	0.1	0.2	0.1	0.0	0.2	0.0	0.0	0.0	0.0	0.0	0.0	0.0	0.2	0.4
Other Bacteria	Planctomycetes 59	0.1	0.0	0.2	0.1	0.0	0.0	0.0	0.2	0.0	0.0	0.0	0.0	0.0	0.0	0.0	0.0	0.0	0.0	0.0	0.3	0.1
Other Bacteria	SAR406 55	0.0	0.0	0.1	0.0	0.0	0.0	0.0	0.2	0.0	0.1	0.0	0.0	0.0	0.0	0.0	0.0	0.0	0.0	0.0	0.2	0.2
Other Bacteria	Tenericutes 1_4	0.4	0.0	0.0	0.7	0.0	0.0	0.0	0.1	0.1	0.0	0.1	0.1	0.1	0.0	0.0	0.0	1.5	0.0	0.0	0.0	0.0
Other Bacteria	Verrucomicrobia 15	0.1	0.0	0.1	0.4	0.0	0.0	0.0	0.2	0.1	0.1	0.0	0.0	0.0	0.0	0.0	0.0	0.0	0.0	0.0	0.3	0.2
